# Microglia Phenotypes in Aging and Neurodegenerative Diseases

**DOI:** 10.3390/cells11132091

**Published:** 2022-06-30

**Authors:** Menbere Y. Wendimu, Shelley B. Hooks

**Affiliations:** Hooks Lab, Department of Pharmaceutical and Biomedical Sciences, College of Pharmacy, University of Georgia, Athens, GA 30602, USA; menbere.wendimu25@uga.edu

**Keywords:** microglia, neuroinflammation, neurodegenerative diseases, Parkinson’s disease, Alzheimer’s disease, hemorrhagic stroke

## Abstract

Neuroinflammation is a hallmark of many neurodegenerative diseases (NDs) and plays a fundamental role in mediating the onset and progression of disease. Microglia, which function as first-line immune guardians of the central nervous system (CNS), are the central drivers of neuroinflammation. Numerous human postmortem studies and in vivo imaging analyses have shown chronically activated microglia in patients with various acute and chronic neuropathological diseases. While microglial activation is a common feature of many NDs, the exact role of microglia in various pathological states is complex and often contradictory. However, there is a consensus that microglia play a biphasic role in pathological conditions, with detrimental and protective phenotypes, and the overall response of microglia and the activation of different phenotypes depends on the nature and duration of the inflammatory insult, as well as the stage of disease development. This review provides a comprehensive overview of current research on the various microglia phenotypes and inflammatory responses in health, aging, and NDs, with a special emphasis on the heterogeneous phenotypic response of microglia in acute and chronic diseases such as hemorrhagic stroke (HS), Alzheimer’s disease (AD), and Parkinson’s disease (PD). The primary focus is translational research in preclinical animal models and bulk/single-cell transcriptome studies in human postmortem samples. Additionally, this review covers key microglial receptors and signaling pathways that are potential therapeutic targets to regulate microglial inflammatory responses during aging and in NDs. Additionally, age-, sex-, and species-specific microglial differences will be briefly reviewed.

## 1. Microglia in Physiology

### 1.1. Microglia Origin, Development, and Maturation

Microglia are resident immune cells of the central nervous system (CNS) with specialized macrophage-like functions. After decades of controversy, a series of parabiosis, transplantation, and lineage tracing experiments have confirmed that microglia and blood monocytes have distinct developmental origins [1,2,3,4] (for historical reviews, see [5]). Unlike circulating monocytes with blood-derived myeloid lineages [6], microglia and most tissue macrophages emerge exclusively from erythromyeloid progenitors in the yolk sac (YS) that differentiate into YS macrophages during embryonic development [2,4]. These precursor YS macrophages migrate to/and colonize the CNS parenchyma prior to the maturation of the blood–brain barrier and subsequently differentiate to microglia [3,4,7,8].

After colonizing the CNS, microglia maintain their population through self-renewal and become ubiquitously distributed in nonoverlapping fields [1,7], accounting for 0.5–16% and 5–12% of total cells in human and mouse brains, respectively [9,10], which varies based on the anatomical region. Microglia maintain a steady number of cells during lifetime through a dynamic and finely regulated balance between local proliferation and apoptosis, without the contribution of peripheral progenitors [11]. According to some studies, microglia exhibit regional heterogeneity within the CNS and are abundant in some regions, including the hippocampus, basal ganglia, and substantia nigra, but are sparse in brain stem cells and cerebellum [9,10]. The relative density of microglia in white and gray matter has been observed to vary between species; white matter of the human cortex exhibits higher microglia density than gray matter [10], while gray matter of the mouse cortex has a higher density of microglia [9]. Dos Santos et al. (2020) recently challenged the idea that microglia have a species-specific distribution, demonstrating that microglia density differs little across different brain structures and mammalian species [12]. These discrepancies have been attributed to the use of different markers to label microglia. The study by Dos Santos et al. used the pan-microglia marker Iba1, while older studies used antibodies that do not stain all microglia populations, such as those that label markers preferentially expressed on activated microglia, including F4/80 [9], or CD68 and MHCII [10].

As microglia mature in the CNS, they exhibit some characteristics that distinguish them from tissue-resident macrophages, such as downregulation of certain cell surface proteins, such as cluster of differentiation 45 (CD45) and MHC class II molecules (MHCII), and expression of unique microglia signature genes, including transmembrane protein 119 (TMEM119), P2Y purinergic receptor 12 (P2RY12), and Sal-like protein (SALL1) [13,14,15,16]. The development, differentiation, and maintenance of microglia in the CNS are regulated by a variety of factors, such as cell–cell interaction of microglia with other cells. The interaction between the microglial CX3-chemokine receptor 1 (CX3CR1) and the neuronal ligand CX3CL1 regulates microglial proliferation and activation [17,18], while interactions between the microglial colony stimulating factor-1 receptor (CSF1R) and neuron-derived secreted ligands, interlukin-34 (IL-34) and CSF-1, are critical for microglial development and survival [19,20,21]. Although they signal through the same receptor, IL-34 and CSF-1 are differentially required for microglia development and maintenance. A developmental study using anti-IL-34 and anti-CSF-1 treatments has shown that microglial colonization in the embryonic brain is dependent on CSF-1, while IL-34 is only required for postnatal microglia maintenance [19]. These ligands also have distinct regional expression; IL-34 is predominantly expressed by neurons in the gray matter and is essential for the development of gray matter microglia, whereas CSF-1 is abundantly expressed by various cell types in the white matter and thus regulates the development of white matter microglia [19]. In addition to neurons, cytokines and chemokines released by other glial cells play a key role in modulating microglial function. For example, astrocyte-derived interleukin-33 (IL-33) promotes the engulfment of microglial synapses during CNS development by signaling through the microglial interleukin 1 receptor-like 1 (IL-1RL1) [22]. Furthermore, various secreted chemokines released by activated astrocytes, such as CCL2 and CXCL10, play an important role in the recruitment of microglia to injury sites [23].

### 1.2. Microglia Functions in Physiology

Microglia are the immune guardians of the brain that play a critical role in providing host defense against pathogens and CNS disorders [24]. Additionally, microglia perform essential housekeeping functions, such as maintaining CNS homeostasis during development, adulthood, and aging [25]. During development, they play an essential role in the regulation of neurogenesis and neuronal survival by phagocytosing newborn apoptotic cells, removing excess synaptic connections, and secreting neurotrophic mediators such as insulin-like growth factor-1 (IGF-1), transforming growth factor- β (TGF-β), and brain-derived neurotrophic factor (BDNF) [26,27]. Microglia are also essential for maintaining brain homeostasis in the adult brain. They provide neuronal support, promote oligodendrocyte development and myelination, phagocytose excess metabolic products and damaged tissues, and play a critical role in learning by regulating synaptic pruning and remodeling of neuronal circuits [28].

### 1.3. Microglia Morphologies

In a healthy brain and under normal conditions, microglia exhibit a ramified morphology with numerous long, thin, and highly branched processes [29]. Ramified microglia were long thought to be in a ‘resting state’ until advances in molecular tools, such as in vivo two-photon imaging, revealed the highly motile nature of microglial protrusions with unique abilities to extend and retract, allowing them to actively survey, detect and respond to environmental aberrations [24,30]. Transcriptome analysis showed that microglia with ramified morphology largely express genes associated with steady-state brain functions such as synaptic integrity, neuronal maturation, and overall maintenance of cell homeostasis [31]. The branched processes of microglia enable them to perform these essential functions, as they allow microglia to constantly interact with neurons and other glial cells, either through direct contact or through secreted mediators [24,30,32]. Upon detecting environmental changes, microglia rapidly migrate toward the triggering stimuli via their branched processes, aided by chemotactic cues [29]. Microglial activation is commonly accompanied by a morphological transformation from a ramified state to an amoeboid state, characterized by an enlarged cell body, shorter processes, and the presence of numerous cytoplasmic vacuoles [24,33,34]. Morphological transformation in activated microglia also accompany functional responses such as migration, antigen presentation, and phagocytosis [31].

Along with the well-known ramified and amoeboid morphologies, other microglia phenotypes have been characterized through ultrastructural studies [35,36]. A bipolar/rod-shaped morphology is described as a transition state between ramified and amoeboid states [37], and this microglia phenotype exhibits distinct transcriptome profiles and high proliferative and phagocytic ability [38,39]. Activated microglia with these morphological features have been characterized mainly in the aged brain and in neuropathological conditions where they closely align with and surround injured axons, generally assuming neuroprotective functions [39,40,41,42]. Additionally, bipolar/rod-shaped microglia are involved in other essential functions, such as synaptic stripping, which supports rewiring of neuronal circuits [37]. Other microglial morphologies have also been reported, including hypertrophic microglia, dystrophic (senescent) microglia, satellite microglia, gitter cell-like microglia, and dark microglia [36].

## 2. Microglia Activation and Polarization

Microglia express various immune pattern recognition receptors (PRRs), including Toll-like receptors (TLRs), nucleotide-binding oligomerization domain (NOD)-like receptors (NLRs), and scavenger receptors (SRs) [43]. PRRs recognize exogenous pathogenic molecules known as pathogen-associated molecular patterns (PAMPs) or endogenous host-derived molecules known as damage-associated molecular patterns (DAMPs) [44,45]. PAMPs and DAMPs have distinct responses; PAMPs induce an antimicrobial response and inflammation in response to infection, while DAMPs drive sterile inflammation, a type of inflammatory response triggered by activation of surface receptors that recognize signals released from damaged cells in response to CNS injuries such as trauma, hypoxia, and NDs [46]. When microglial PRRs interact with PAMP/DAMPs, a variety of intracellular cascades, kinases, and downstream transcription factors are activated, ultimately leading to the synthesis of molecular mediators of inflammation and other cellular responses [45,46].

**Figure 1:**
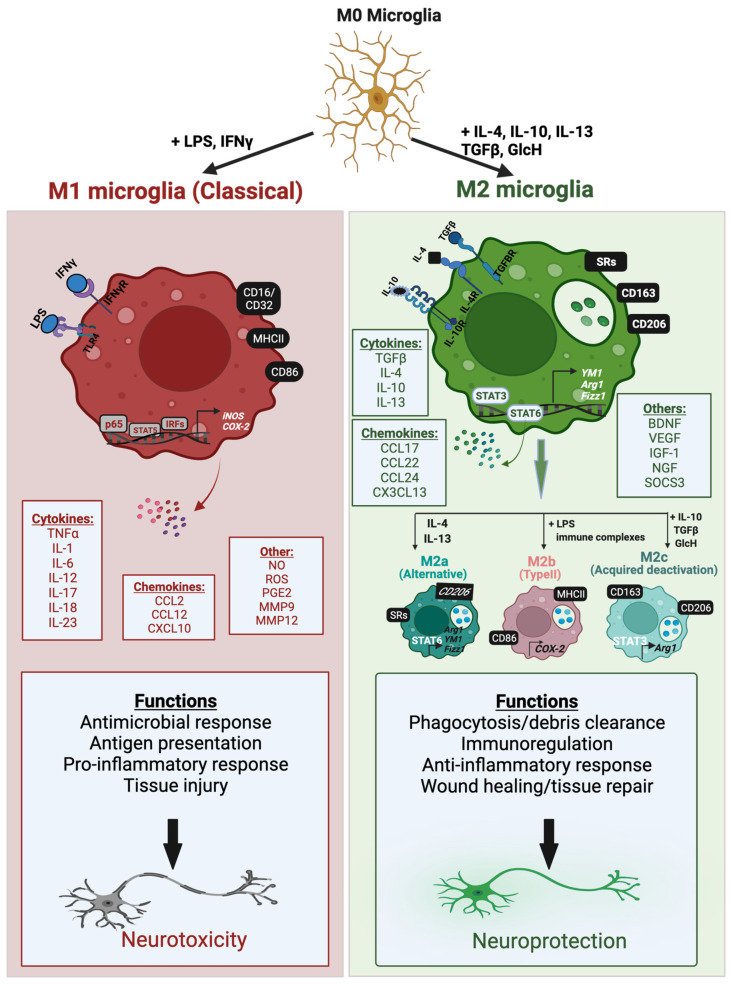
Microglial activation phenotypes and functions. Microglia can be polarized from a resting state (M0 microglia) to two main activation phenotypes, classically activated M1 microglia and alternatively activated M2 microglia, which are induced by interaction of inflammatory molecules with their respective cognate receptors. These microglia phenotypes exhibit a variety of phenotypic markers that are expressed intracellularly or on the cell surface, as well as molecules that are secreted from the cell, such as chemokines, cytokines, and other effector molecules. The M2 phenotype is further subdivided into three subtypes: M2a (alternatively activated state), M2b (type II activation phenotype), and M2c (acquired deactivation state). The M2b subtype shares similar features as M1 microglia and is induced by inflammatory molecules. The phenotypes of M1 and M2 microglia perform distinct functions that can be neurotoxic or neuroprotective, respectively. Figure created using BioRender.com (accessed on 14 April 2022).

Microglia activation is associated with changes in the expression of cell surface receptors, unique polarization responses, and the release of a variety of inflammatory mediators that contribute to either a tissue reparative protective role or a detrimental neurotoxic response. Although oversimplified, microglial activation is frequently defined in terms of two broad polarization states: a classically activated M1-like phenotype or an alternatively activated M2-like phenotype. These polarization states differ in the triggering stimuli, expression of phenotypic markers and secreted mediators, all of which determine the overall outcome. In general, the classically activated M1 phenotype is associated with pro-inflammatory and neurotoxic responses, while the M2 phenotype mostly mediates anti-inflammatory and neuroprotective functions [47] (Figure 1).

The M1-like microglia phenotype is induced in vitro by interferon-γ (IFNγ) and/or the Gram-negative bacterial endotoxin lipopolysaccharide (LPS). LPS is a ligand for Toll-like receptor 4 (TLR4) which couples with coreceptors to activate various pro-inflammatory transcription factors including NFκB, AP1, STAT5 and IRFs, via TIR domain-containing adapter inducing IFNβ (TRIF) and myeloid differentiation primary response protein 88 (MyD88)-dependent pathways [48,49]. IFNγ acts on IFNγ receptors 1 and 2 (IFNγR 1/2) and activates the JAK/STAT pathway that leads to the phosphorylation and nuclear translocation of STAT1 and other IRFs [50]. Transcription factors activated by M1-like microglia trigger upregulation of pro-inflammatory cell surface markers, such as MHCII and the cluster of differentiation marker 86 (CD86) [29,51] Additionally, they induce the production of a variety of pro-inflammatory mediators, including cytokines, such as tumor necrosis factor-α (TNFα) and interleukins (IL-1β, IL-6, IL-12, IL-17, IL-18, IL-23), chemokines such as CCL12 and CXCL10, and other pro-inflammatory mediators, including reactive oxygen and nitrogen species (ROS and RNS), inducible nitric oxide synthase (iNOS) and cyclooxygenase-2 (COX-2) [51,52,53,54]. M1-like microglia play an important role in eliciting innate immune responses to combat foreign pathogens and trigger the adaptive immune response [55]. However, chronic activation in pathological conditions contributes to neuroinflammation, oxidative stress, and neurotoxicity [55,56] (Figure 1; left side).

M2 polarized microglia can assume an ‘alternatively activated’, or ‘acquired deactivation’ state, and are often associated with functions such as immune resolution and tissue repair through the secretion of anti-inflammatory and neurotrophic factors [47,56,57]. When brain homeostasis is disrupted due to brain injury or chronic stress, the CNS has endogenous defense mechanisms that promote tissue repair. Various anti-inflammatory cytokines, growth factors, and hormones, such as glucocorticoids, are released by injured neurons and promote surrounding microglia to transform into an M2-like protective phenotype [58,59]. M2 microglia can be activated by four main anti-inflammatory cytokines, IL-4, IL-10, IL-13, and TGF-β. IL-4 and IL-13 promote the alternative activation state and generally function to antagonize M1 pro-inflammatory responses, such as the production of TNFα, IL-6, and iNOS [60,61]. The multifunctional cytokine TGF-β plays a pivotal role in angiogenesis, immunoregulation, and tissue repair, and together with IL-10, induces the acquired deactivation state [47]. The M2 phenotypes are subclassified into three states, M2a, M2b, and M2c, which have overlapping biochemical roles but differ in the activating stimuli, marker expression, and mechanism of actions [53]. (Figure 1; right side).

M2a is considered an anti-inflammatory, phagocytic, and wound healing phenotype, and is activated upon stimulation with IL-4 or IL-13 cytokines [56]. This is accompanied by the activation of the JAK1/3-STAT6 pathway leading to upregulation of cell surface markers CD206 (mannose receptor), Arg1 (arginase-1), YM1 (chitinase-like protein), Fizz1 (found in inflammatory zone 1), and different scavenger receptors (SRs) [29,52]. The M2a phenotype is involved in immunity against parasites, collagen formation, and tissue repair [51]. M2b microglia, also known as type II, are generally characterized as an inflammation regulatory phenotype and are activated by fusion of TLR and FCγ receptors, and subsequent interaction of these receptors with B cell-derived IgG [62,63,64]. M2b polarized microglia share similar characteristics as M1 microglia in that they can be activated in vitro by TLR agonists and are characterized by the expression of COX-2 and M1-associated cell surface proteins CD86 and MHCII [65,66]. However, M2b microglia also have different responses, such as the recruitment of regulatory T cells and the release of the anti-inflammatory cytokine IL-10 [65]. IL-10, along with TGF-β and glucocorticoids, triggers activation of the immunosuppressive M2c phenotype [51,67]. M2c polarized microglia exhibit an ‘acquired deactivation’ state and play prominent roles in matrix remodeling, tissue repair, and immunoregulation [29,51]. IL-10-induced M2c polarization is mediated by interaction with IL-10R1 and IL-10R2 receptors and subsequent activation of the JAK1/STAT3 pathway. This leads to upregulation of the cell surface marker CD163, and TGF-β and IL-10 cytokines, and inhibition of M1-associated pro-inflammatory cytokines [51,53].

Microglia are capable of dynamically shifting polarization states between M1-like and M2-like phenotypes. Rather than employing two distinct activated states, M1 and M2 represent a continuous spectrum of various activation phenotypes, and different phenotypic markers can coexist together, suggesting several intermediate phenotypes [56]. Advances in genome-wide expression profiling studies have further elucidated the biological complexity of microglial polarization response in which mixed phenotypes are evident with simultaneous expression of M1 and M2 markers, prominently in aging and pathological disease models [68,69,70]. For example, the presence of a mixed transitional phenotype known as Mtran has been demonstrated in a traumatic brain injury (TBI) model in which a significant proportion of TGF-β (M2 marker)-positive microglia also coexpressed the M1 marker CD16/32 [71]. An intermediate polarization state termed ‘M1 ½’ has also been reported in rd1 retinal degeneration mouse model [72]. Recently, an atypical M2d phenotypic state has been described which is induced by transforming the M1 phenotype into a pro-angiogenic/anti-inflammatory M2 activated phenotypic state, and this phenotypic switch is mediated by coactivation with TLR ligands and the adenosine A2A receptor (A2AR) [73,74]. The M2d polarization phenotype is explored primarily in tumor-associated macrophages and its significance in microglia is unclear.

The highlighted studies demonstrated that the oversimplified M1/M2 paradigm, which was largely understood through in vitro assays, does not accurately model the complexity of microglia phenotypes in vivo, where the response of microglia is dictated by intricate interaction with the brain microenvironment. However, the broad binary characterization of microglia phenotypes is referenced in this review, as it provides insight into the pro-inflammatory and anti-inflammatory nature of microglia with beneficial and neurotoxic functions. The overall contribution of microglia in the normal and diseased brain and the transition between different phenotypic states depend on the activating mechanism, the duration of the signal, and the regulatory signaling molecules [75,76]. A select few modulatory factors are discussed in Section 7.

## 3. Microglia Phenotypes in Aging

Compelling evidence suggests that many NDs are characterized by preclinical progression, whereby age-associated pathological alterations occur at the cellular and molecular levels several years before clinical diagnosis [77]. In recent years, there has been increasing interest in identifying aging-associated changes in microglial activation, function, and molecular signatures. There are limited aging studies in human microglia, as there is shortage of postmortem human brain samples and a lack of noninvasive imaging and genetic manipulation tools to visualize and characterize live human microglia. Additionally, aging research is expensive and time-consuming due to the long waiting period for the natural aging process to take place in preclinical models. Therefore, despite recent advances in transcriptomics and live imaging methodologies [78,79], our current understanding of microglial responses in aging is largely derived from studies using transgenic model organisms with accelerated aging to mimic the natural process of aging.

**Figure 2:**
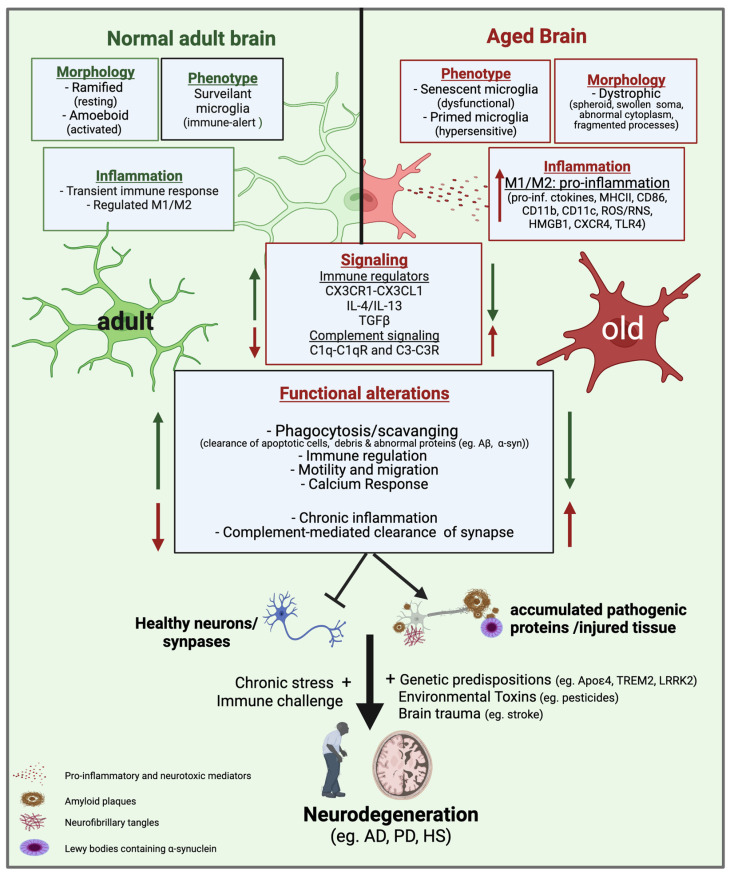
Age-dependent microglial changes implicated in neurodegenerative diseases. During aging, microglia exhibit alterations in their morphology, phenotypes, inflammatory response, and overall functional response. Healthy adult microglia with normal branched processes can effectively survey the brain microenvironment, and upon encountering infections, injury, and inflammatory agents, they change morphology and execute effector functions by becoming transiently activated. In comparison, old microglia have a dystrophic morphology associated with cellular senescence and exhibit a primed phenotype in which they are hypersensitive to environmental changes and produce large amounts of M1-like pro-inflammatory and neurotoxic mediators which contribute to chronic neuroinflammation. Age-associated loss of endogenous microglial regulatory pathways, such as CX3CR1-CX3CL1 signaling and TGF-β signaling, impairs the ability of microglia to regulate inflammatory responses. Aging is also associated with a decline in calcium response, motility, and phagocytosis functions, all of which impair microglial clearance of injured cells, tissue debris, and pathogenic proteins. However, complement-mediated phagocytosis mechanisms are upregulated in aged microglia and contribute to the loss of healthy neurons. These age-related microglial dysfunctions, combined with a chronic stress environment, brain trauma, or genetic predisposing factors, can contribute to the progressive onset of neurodegenerative diseases. AD: Alzheimer’s disease; PD: Parkinson’s disease; HS: Hemorrhagic stroke. Figure created using BioRender.com (accessed on 14 April 2022).

Growing evidence suggests that during aging, microglia undergo a morphological transition from a ramified state to a spheroid-activated phenotype with abnormal cytoplasmic structure and fragmented processes associated with microglial dystrophy [80,81,82]. In vivo positron emission tomography (PET) imaging of human brains using the PET ligand (R)-[11C]PK11195 showed a widespread distribution of activated microglia in human cortical and subcortical regions during healthy aging [83]. Aged microglia also exhibit an inflammatory hypersensitive phenotype, often referred to as ‘primed microglia’ [84,85]. Primed microglia are overreactive to inflammatory and neurotoxic insults and produce large amounts of pro-inflammatory cytokines, chemokines, and reactive species [84] (Figure 2).

Some studies have also examined microglial polarization responses associated with age. Recently, Wang et al. (2019) compared microglial M1/M2 markers in 2, 6, 18 and 28-month-old rat brains, and observed an age-dependent increase in transcript and protein levels of M1 markers (TNFα and IL-1β) and an opposite trend in M2 marker expression (Arg1 and IL-10) [86]. The observed increase in M1/M2 marker ratio also correlated with age-induced dopaminergic (DA) neuronal loss. Relative loss of the M2-like phenotype has also been reported in aging mice, as demonstrated by suppressed anti-inflammatory IL-4/IL-13 signaling [87]. Increased M1-like microglia responses such as upregulation of TLRs, various activation markers (MHCII, CD68, and CD86), and microglia/macrophage-specific inflammatory receptors CD11b are also evident in aged brains of rodents, canines, humans, and non-human primates [80,84,88,89,90,91,92]. These results suggest that aged microglia have a predominant M1-like phenotype associated with neurotoxic responses.

Age-associated loss of endogenous microglia regulatory pathways has been implicated as a mechanism for the presence of reactive microglia in the aged brain. For example, impaired TGFβ-signaling, a pathway that promotes microglia quiescence, has been reported in the aged brain and is associated with a reduction in the protective function of microglia [93,94]. Furthermore, downregulation of microglial receptors involved in microglia-neuron interactions is prominent in the aged brain, such as the P2Y purinergic receptor 12 (P2Y12R), which is a key regulator of microglial activation and phenotypic transformation [95]. Similarly, age-related neurodegeneration leads to the loss of neuron-derived immunomodulatory molecules, such as the CX3CL1 ligand, which keeps microglia in a quiescent state [96]. These alterations in endogenous regulatory factors contribute to chronic activation, microglial dystrophy, and neurodegeneration (Figure 2).

Transcriptome studies have allowed researchers to characterize global changes in gene expression associated with aging. A recent postmortem study by Soreq et al. (2017) analyzed the transcriptome profiles of glial and neuronal cell types in different regions of the human brain in 480 subjects aged 16 to 106 years and observed an increase in microglia-specific genes in all regions of the brain that strongly predict biological aging, such as upregulation of complement molecules and inflammatory responses [97]. A separate study also identified signature genes involved in normal aging, such as high expression of genes encoding TNF family ligands, vesicle release proteins, and the pro-inflammatory cytokine high mobility group box 1 (HMGB1) [98]. HMGB1 mediates microglia priming in aged brain, and its inhibition is known to desensitize aged microglia to an inflammatory insult [99].

A transcriptomic coexpression meta-analysis by Holtman et al. (2015) also examined changes in microglia signatures in four mouse models of aging and NDs [85]. They compared normal aged mice, Ercc1ko-accelerated aging mice with loss of DNA repair mechanism, and transgenic models of Alzheimer’s disease (AD) and Amyotrophic Lateral Sclerosis (ALS). Their study identified common microglial inflammatory gene networks shared across normal aging and in pathological models that mediate age-associated microglial priming. Unlike LPS-induced upregulated acute inflammatory gene networks, such as activation of NFκB signaling, primed microglia of these aging models displayed upregulated expression of MHCII and other pro-inflammatory genes encoding cell surface markers CD11c integrins and CXC-chemokine receptor 4 (CXCR4). Most of these signature primed microglia gene networks were involved in functions related to lysosome, phagosome, oxidative phosphorylation, and antigen presentation, indicating that aged microglia have impaired immune regulation and phagocytosis response.

In a healthy developing brain, one of the main housekeeping functions of microglia is the pruning of synapses through a phagocytic clearance process. This is mediated primarily through a mechanism involving the recognition of complement proteins on synapses via microglial complement receptors [100]. However, complement-mediated clearance mechanisms are known to normally be lost in healthy adult microglia, but later reemerge during the aging process, leading to a dysfunctional microglial phenotype that can contribute to synaptic loss and neurodegeneration [101] (Figure 2). For example, complement proteins C1q and C3 are evident in the aging brain and are deposited on synapses and can activate phagocytic complement receptors C1qR and C3R expressed in microglia and then trigger the clearance of healthy synapses [102,103,104,105]. These findings implicate a detrimental role for complement-mediated phagocytosis in aging that can contribute to neurodegeneration.

Age-related functional change in the brain is also associated with dysregulation of calcium signaling [106,107], which can lead to a variety of neuropathological conditions. Calcium homeostasis is impaired in the aged brain due to age-dependent deregulation of important calcium channels and dysfunction in mitochondria and the endoplasmic reticulum (ER) [107]. Microglia express various plasma membrane ionotropic and metabotropic receptors that are coupled to changes in intracellular calcium levels [Ca^2+^]_i_, including two main classes of nucleotide receptors, purinergic P2X receptors and P2Y receptors [108,109]. Changes in Ca^2+^ response triggered either by activation of these receptors or indirectly by other signaling pathways play an essential role in regulating various executive functions of microglia, such as phagocytosis and inflammatory responses [110,111]. Visualization of live microglial Ca^2+^ dynamics is increasingly being used to study functional and morphological changes in the intact brain [106,112,113]. Recently, Olmedillas del Moral et al. (2019) used high-resolution two-photon microscopy and in vivo Ca^2+^ imaging to compare changes in the functional properties of cortical microglia in three different cohorts of mice: young (2–4 months old), middle-aged (9–11 months old), and old (18–21 months old) [106]. Their study revealed two distinct phenotypes of aging microglia. Middle-aged microglia exhibited a reactive ‘immune-alert’ phenotype, characterized by normal process motility and increased spontaneous [Ca^2+^]_i_ signaling, while microglia from older mice showed a reduced Ca^2+^ response and disorganized motility, resembling a dysfunctional/senescent phenotype. In addition to alterations in Ca^2+^ responses in the aging brain, Ca^2+^ dysregulation is also prominent in response to injury, inflammation, and neurodegenerative diseases [114,115]. In general, the evidence reviewed here suggests that age-associated impairment of microglial responses throughout life contributes to the development of neurodegenerative diseases (Figure 2).

## 4. Microglia in Acute and Chronic Neurodegenerative Diseases

This section will review the complex microglial phenotypes and functions in three neurodegenerative diseases, hemorrhagic stroke, Alzheimer’s disease, and Parkinson’s disease, focusing on the specific microglial response shaped by the underlying pathology.

### 4.1. Microglia in Hemorrhagic Stroke

#### 4.1.1. Hemorrhagic Stroke (HS)

Hemorrhagic stroke (HS) is primarily caused by a rupture of blood vessels that results in bleeding within or surrounding the brain parenchyma [116,117]. There are two main subtypes of HS: intracerebral hemorrhage (ICH-within the brain) and subarachnoid hemorrhage (SAH-surrounding the brain). HS is known for its high mortality and morbidity, with more than 50,000 annual deaths reported in the United States [118]. In particular, ICH has the highest mortality rate: approximately 30–50% of patients die within the first month, and most surviving patients experience long-term disability [117,118]. The incidence of HS increases with age, as the two main predisposing factors, chronic hypertension and amyloid angiopathy, are more prominent in the elderly [118,119].

Although HS is considered a cerebrovascular disease, it can cause acute tissue destruction, hematoma formation, and elevated intracranial pressure, which can lead to brain damage [120,121]. Immediate neuronal death in the acute phase of injury subsequently triggers secondary brain damage. Currently, there is no cure for hemorrhagic stroke and treatment options are limited primarily to supportive care [122,123]. Therefore, there is an urgent need to characterize the molecular mechanism of HS and identify therapeutic targets underlying early and delayed brain injury. In HS, one of the key players in secondary neurodegeneration is chronic neuroinflammation, and therefore promising immunotherapeutic approaches have been considered to mitigate inflammation-induced neurodegeneration [124].

#### 4.1.2. Microglia Activation in HS

Microglia and infiltrating macrophages are one of the first immune respondents after ICH, and thus play a fundamental role in disease progression [125]. In the hemorrhagic brain, numerous molecular mediators trigger microglial activation, including complement components, coagulation factors, and blood-derived hematoma products [126,127]. Microglia activation is a prominent feature of HS, and its activation response has been studied in patients and experimental models. Studies in a collagenase-injection ICH model demonstrated increased in microglia/macrophage population in the acute stage of injury (days 1–3) and a gradual decline to baseline levels after 21 days [128,129]. Activated microglia populations were observed as early as 1 h after ICH and this activation response is prominent in the perihematomal region surrounding the injury site [130]. In an autologous blood injection model of ICH, microglia activation is evident beginning at 4 h after insult and persisted up to 4 weeks, with a peak density observed 2–3 days after injury [126,131,132]. A contrasting finding using the same ICH model showed a delayed peak in maximal activation that occurs in the subacute stage of injury around days 7–10 (d7–10) [133]. Although the use of specific microglial markers and experimental models has led to discrepancies in observations, collective evidence suggests an early microglial recruitment and activation response in HS that changes throughout the course of the disease.

#### 4.1.3. Microglia Activation by Hematoma Components

HS induces the lysis of red blood cells (RBCs) and the release of hemoglobin, accompanied by the subsequent production of free heme [126,127]. In the brain, elevated concentrations of hemoglobin, heme, and its oxidized form hemin trigger brain injury by inducing microglial activation, neuroinflammation, oxidative stress, and edema formation [121,127,134,135]. Heme metabolism in microglia plays a diverse role in HS. Phagocytic microglia can help sequester large amounts of extracellular heme [136,137,138], thereby limiting its neurotoxic effect to other cell types. However, uptake of excess heme/hemin can cause accumulation of toxic heme degradation products in microglia that can exacerbate HS injury [127,134]. Hematoma components modulate microglia activity by activating specific receptors and inflammatory pathways. In particular, heme-induced inflammatory damage is believed to occur primarily through TLR4, and this receptor is upregulated in microglia in response to ICH and exogenous heme treatment [139,140] (Lin et al., 2012; Teng et al., 2009). Heme-induced TLR4 activation, in turn, activates NFκB and promotes pro-inflammatory signaling via MyD88/TRIF pathway [140].

After brain hemorrhage, heme processing enzymes are upregulated in microglia, including heme oxygenase-1 (HO-1), which is an inducible rate-limiting enzyme that converts heme/hemin to carbon monoxide, biliverdin, and ferrous iron [130,141]. Since HO-1 exerts its effect through these various metabolites, its exact role after ICH is not clear. Carbon monoxide and biliverdin mainly mediate antioxidant and neuroprotective effects, while ferrous iron is largely associated with tissue damage [142]. Like heme, iron can induce microglia activation through the TLR4/Myd88/TRIF pathway [139]. After ICH injury, HO-1 is induced in both microglia and astrocytes, and its expression changes during disease progression [143]. Recent studies showed that HO-1 exerts its neurotoxic effects in the early stages of injury, at which time its expression is higher in microglia, while it primarily mediates its neuroprotective functions during the recovery stage when it is predominantly expressed in astrocytes [143,144,145]. These findings implicate microglial HO-1 as a neurotoxic mediator in the early phase of cerebral hemorrhage. Upregulation of HO-1 in microglia can lead to iron overload and exacerbate hemorrhagic brain injury by contributing to further activation of microglia, oxidative stress, brain edema, and neuronal death [142]. In support of this, several studies have shown that selective ablation of HO-1 in the early stages of injury (1–3 days post ICH) or the use of iron chelating agents 2–12 h post ICH improves functional recovery, and their protective effects are partly mediated in part by regulation of microglial activity [130,146,147]. Overall, the evidence presented in this section suggests that hematoma-induced activation of microglia and subsequent induction of HO-1 accelerate hemorrhagic brain injury by promoting neuroinflammation, oxidative stress, and iron toxicity.

#### 4.1.4. Microglia Activation by Thrombin

Microglia are also activated following ICH by the serine protease thrombin, which acts as a blood coagulation factor. Thrombin, normally present in blood plasma, is introduced into the brain immediately after brain hemorrhage or after BBB disruption [127]. Thrombin accumulation in the brain exacerbates edema formation, inflammation, and neurodegeneration, partly due to its role in inducing microglial activation [148,149,150]. Several in vivo and in vitro reports have shown that thrombin-mediated microglia activation results in upregulation of M1-associated inflammatory mediators such as iNOS, NO, COX-2, MHCII, and pro-inflammatory cytokines, including IL-1β, IL-6, and TNFα [151,152,153,154,155].

The effect of thrombin on microglia activation and the pro-inflammatory response is primarily attributed to downstream signaling mediated by protease-activated receptor (PAR) activation, which are G protein-coupled receptors expressed in various cells, including microglia. In particular, thrombin activates microglia by modulating several intracellular signaling mediators such as PKC (Ryu et al., 2000), NFκB (Ryu et al., 2000), p38 and p44/42 (ERK1/2) (Suo et al., 2002; Choi et al., 2003; Ohnishi et al., 2012), and JAK2-STAT3 [152,153,154,155]. Prominently, MAPK signaling pathways have been suggested to be an important mechanism underlying thrombin-induced microglial activation and ICH injury [156,157].

The member of the prototypical PAR family, PAR1, is strongly expressed on the surface of microglia/macrophages after CNS injury and is highly sensitive to thrombin stimulation [158]. PAR-1 activation contributes to ICH-induced brain injury by enhancing neuroinflammation, brain edema, DNA damage, and neurotoxicity [149]. PAR-1 activity also modulates microglia proliferation, activation, and polarization responses [151,155,159,160]. In the experimental ICH model, PAR-1 is activated after ICH injury and its activation coincides with the timing of microglia/macrophage activation and polarization, and its deficiency suppressed M1 phenotype activation and the production of pro-inflammatory cytokines [160]. These findings implicate PAR-1 as a central player in thrombin-induced microglial activation and polarization in HS.

Thrombin can also modulate microglia activation independently of PAR-1. In cultured rodent microglia cells, thrombin induces transient Ca^2+^ release from intracellular stores and promotes the production of NO and pro-inflammatory cytokines [161]. This effect of thrombin was shown to be mediated by its enzymatic activity rather than its effect on PAR activation. A potential receptor-independent mechanism for thrombin-induced ICH injury is the cleavage of complement proteins leading to complement activation, which plays an essential role in microglia activation and neuroinflammation [162,163]. In support of this, complement proteins C3a and C5a have been linked to microglia activation after ICH [116]. Perihematomal microglia activation and subsequent ICH damage are significantly reduced in complement C3-deficient mice [164]. In the absence of complement C3, complement activation can be triggered by complement C5a [165]. The C5a receptor is upregulated in microglia after ICH, and its inhibition suppresses the expression of pro-inflammatory M1 markers, such as iNOS, TNFα, IL-1β, and IL-6 [166]. In general, these studies highlight the diverse role of thrombin in modulating the response of microglia in HS. Additional studies should characterize the PAR-1 dependent and independent mechanisms underlying thrombin-induced microglial activation response, which will help to specifically identify detrimental targets in HS.

#### 4.1.5. M1 and M2 Microglia in HS

Although microglia activation is triggered by thrombin and hematoma component, the nature of the activation state changes throughout the course of the disease, and microglia assume different phenotypic states. Several studies have explored the role of different microglia phenotypes in the context of HS, and collective evidence suggests that M1-like microglia primarily mediate neurotoxic effects by enhancing neuronal apoptosis, brain edema, and blood–brain barrier permeability [116,167]. Receptors that trigger M1 polarization, such as TLR4, have been shown to be upregulated in hemorrhagic patients and are associated with neuroinflammation, oxidative stress, and poor functional outcomes [139,168,169,170,171].

Very little is known about the function of M2-like anti-inflammatory microglia in HS. However, findings from a few studies suggest that alternatively activated M2-like microglia exhibit neuroprotective functions. Specifically, various molecular mediators that trigger M2 polarization, including TGF-β, peroxisome proliferator-activated receptor gamma (PPARγ), and anti-inflammatory cytokines (IL-4 and IL-10), have protective functions after ICH and SAH injury due to their role in wound healing and resolution of inflammation [172,173,174,175]. The M2 activation state is also associated with an enhanced phagocytosis response due to higher expression levels of scavenger receptors, including CD36, CD206, and the hemoglobin-scavenger CD163 (hemoglobin and CD206 [137,172,176]. Therefore, M2-like microglia serve a protective function in HS by phagocytosing hematoma products and removing cellular debris.

Overall, the presented findings suggest that immunotherapies that modulate the phenotypic switch from neurotoxic (M1-like) to neuroprotective (M2-like) state are promising candidates for the treatment of HS. Since the stage-specific transition of microglia phenotypes impacts the overall function of microglia, a thorough characterization of time-dependent microglia activation response is needed to identify an optimal therapeutic time window for targeting microglia.

#### 4.1.6. Temporal Microglia Polarization in HS

Existing evidence suggests that microglia phenotypes exist in a spectrum following ICH injury with both M1 and M2-like phenotypes found in the peri-hematoma region throughout the course of ICH [177]. However, the M1/M2 proportion changes in which M1-like microglia response is thought to predominate in the early phase of hemorrhagic injury followed by a switch in balance towards M2-like phenotype in the subacute stage [125,178]. This M1 to M2 trajectory is supported by microscopy-based preclinical studies in ICH and SAH models (ICH and SAH models) [160,173,179,180,181]. In both autologous blood-injection and collagenase-injection models of ICH, M1-specific phenotypic markers are upregulated immediately after injury. A peak in M1 marker expression is evident within the first few hours of injury and remained high after 72 h, gradually returning to baseline on day 7 [116,160,182]. Specifically, known pro-inflammatory cytokines such as IL-1β, IL-6, and TNFα are elevated in perihematomal tissue as early as 6 h after ICH, and expression levels of these markers increase within the first 3 days [181]. In addition to cytokines, transcript levels of prominent M1 surface markers, such as CD32, CD68, and CD86, are acutely upregulated and remained elevated after 3 days [180]. A notable increase in CD16/CD32-expressing microglia is also evident in the perihematomal region within the first few days [181], and acutely upregulated pro-inflammatory proteins such as CD16 and iNOS decline in expression a week after injury [160]. Data in SAH experimental models also demonstrated a similar trend of early-phase M1 polarization response post-injury [167,179]. Overall, these results implicate a detrimental role for M1 microglia in the early stage of hemorrhagic injury.

Unlike rapidly induced M1 markers, M2 phenotype markers gradually increase during HS progression [116,160,167,173,179,180]. However, upregulation of M1 markers does not always precede M2 phenotypic markers, as evidence has shown mixed expression of M1-like and M2-like phenotypic markers in the acute stage of injury (days 1–3) [179]. Nonetheless, in both subtypes of HS, the peak in the expression of M2 markers appears to be delayed compared to M1 markers [116,167,181] (Li et al., 2018; Wan et al., 2016). For example, Lan et al. (2017) demonstrated that, while there are microglia populations expressing both CD16/CD32 and YM-1 in the perihematomal region at days 1 and 3 post-ICH, the proportion of YM1^+^-M2-like microglia was higher at 72 h compared to CD16/CD32 positive cells [181]. Studies in the SAH model also showed that anti-inflammatory cytokines expressed by M2-like microglia cells, such as IL-4 and IL-10, show a relatively gradual increase following the acute pro-inflammatory response [179].

Accurate characterization of the dynamic microglial activation and polarization response has not been possible since interpretations in different studies are based on a few molecular markers. For example, Yang et al. (2016) showed steady expression of the M1 markers CD68 and IL-1β in activated CD11b+ microglia/macrophage cells at different stages of the disease, ranging from 1 to 28 days after collagenase-induced ICH injury [173]. This expression pattern is not consistent with other studies and does not support the notion that microglial M1 response declines over time. To comprehensively examine the temporal activation of microglial phenotypes, recent bioinformatics studies have employed tools that capture a wide variety of signature markers. Taylor and colleagues (2017) incorporated flow cytometry and transcriptome analysis to characterize the M1/M2 microglial response in the acute and resolution phase of injury in a blood-injection ICH model [175]. Their findings showed that microglia in perihematomal tissue exhibit a transient pro-inflammatory phenotype in the acute phase (day 1) that was suppressed on day 7, at which point the microglia transitioned to a type of alternative activation state. Multiplexed ELISA analysis of perihematomal tissue samples also revealed a phenotypic transition from M1 to M2-like microglia within the first 2 weeks after ICH, further suggesting that the balance of M1/M2 decreases throughout the course of disease. Interestingly, their findings demonstrated that the phenotypic switch during the resolution phase of ICH does not reflect changes in anti-inflammatory signature cytokines such as IL-4 and IL-13, and instead involved upregulation of alternative activation phenotype genes associated with wound healing and brain recovery functions, such as TGFβ-1 signaling pathway [175]. These findings shed light on the complex nature of microglial polarization response after brain hemorrhage.

The dynamic expression pattern of inflammatory cytokines and surface proteins varies in different experimental models of ICH [116]. For example, the switch from M1 to M2-like phenotype is reported to be delayed (day 7) in a blood-induced ICH model [175], compared to collagenase injection models (days 1–3) [181]. This difference has been attributed to the greater disruption of the BBB that occurs in collagenase-induced ICH injury, which enhances the infiltration of peripheral immune cells [183]. Therefore, the increased infiltration of M2-like peripheral macrophages through the leaky BBB may explain the accelerated accumulation of M2 phenotypes in the collagenase model of ICH.

Limited studies have analyzed polarization markers in clinical samples from HS patients. Consistent with findings in experimental models, the expression level of TLR4, which is associated with the M1-like pro-inflammatory response, is upregulated in the early phase of injury in peripheral macrophages isolated from SAH patients, and the expression level declined in the subacute phase, reaching normal levels on day 7 [168]. On the contrary, the M2 phenotypic marker TGF-β1 is suppressed in the acute stages of injury in plasma samples obtained from ICH patients, and a lower TGF-β1 level in the early phase of injury correlates with poor functional recovery [175]. Thus, while there are mixed M1/M2 phenotypes after brain hemorrhage, a balance of evidence suggests a neurotoxic role for M1-like microglia in the early phase of hemorrhagic injury and a protective role for M2 microglia in the late phase recovery process. Given the limitations of existing animal models and experimental approaches, further clinical investigations are needed to better characterize the dynamic response of microglia phenotypes in HS.

### 4.2. Microglia in Alzheimer’s Disease

#### 4.2.1. Alzheimer’s Disease (AD)

Alzheimer’s disease (AD) is the most prevalent age-related neurodegenerative disease characterized by a progressive loss of neurons in the cerebral cortex, leading to cognitive impairment, executive dysfunction, behavioral deficits, and dementia [184]. Although age is the main risk factor, various mechanisms contribute to the onset and progression of AD, making it one of the most complex NDs. AD has two main subtypes, which differ according to genetic predisposition and age of onset [185]. Familial AD (fAD) is a less common form of AD that commonly manifests its symptoms before the age of 65 years and has a mendelian inheritance pattern with minimal influence from the environment. Sporadic AD (sAD) is the most prevalent form of AD after the age of 65, and its etiology involves a combination of genetic, environmental, and lifestyle factors that contribute to age-associated neuropathological changes [185]. Neuropathological hallmarks of AD include loss of neurons and synapses in the cortical and subcortical brain regions, accompanied by extracellular accumulation of amyloid-β (Aβ) plaques and intraneuronal aggregation of hyperphosphorylated tau protein (pTau) known as neurofibrillary tangles (NFTs) [186].

Aβ is a 37–49 amino acid residue peptide that is produced through proteolytic processing of amyloid-β precursor protein (APP) by sequential cleavage involving β-secretase and *γ*-secretase enzymes [187,188]. Aβ peptides are capable of self-assembling and transforming to soluble oligomeric or insoluble heavy aggregate with varying neurotoxic potential [189]. Various genetic risk factors of AD are linked to Aβ accumulation in the CNS, which is often associated with a defect in Aβ clearance mechanisms [190]. Prominent examples include mutations in genes encoding APP or presenilin 1/2, which are catalytic proteins involved in APP breakdown and the release of Aβ [191]. Furthermore, the presence of the apolipoprotein ε4 allele (APOE4) is one of the main genetic risk factors for AD, and this protein plays a central role in the regulation of lipid homeostasis and Aβ accumulation in the brain [192].

Aβ accumulation in the brain has been proposed as a potential trigger for AD and has formed the traditional ‘amyloid hypothesis’, which suggests that a defect in Aβ metabolism in the aged brain leads to a linear sequence of events involving the accumulation of toxic Aβ and the formation of amyloid fibrils that subsequently develop into senile plaques and trigger tau pathology and neurotoxicity [193]. This hypothesis led to the development of several Aβ-modifying therapeutic strategies that target the production or aggregation of Aβ or promote its degradation. In June 2021, the FDA approved aducanumab antibody, which degrades toxic Aβ forms for the treatment of AD [194]. However, its approval has been controversial, as clinical trials showed little or no functional improvement in AD patients, and the use of aducanumab is now only recommended for patients with mild cognitive impairment (MCI) or mild dementia due to AD [195]. Therefore, despite decades of drug discovery efforts, there is currently no effective treatment to cure AD, and several other clinically tested disease-modifying agents have failed to mitigate its progressive symptoms. Therefore, there is a growing need to identify preventive approaches that mitigate the onset of AD. This requires a better understanding of the underlying mechanism of AD pathology.

#### 4.2.2. Microglia Activation in AD

Genome-wide association studies (GWAS) have implicated a significant role for microglia in AD pathogenesis [196]. Variants of highly expressed microglial transcripts are identified as risk factors for AD, and these AD-associated susceptibility genes strongly affect microglial function. For example, one of the major risk factors associated with AD is mutations in TREM2, a prominent microglial receptor involved in modulating microglial activation and phagocytosis response [197]. Furthermore, several other risk factors for AD have been identified, such as alterations in the microglial genes CD33, MS4A6, and ABCA7 [196], which mediate important functions of microglia, further suggesting an important role for microglia in AD pathogenesis.

In vivo PET imaging studies have shown that microglia activation occurs early before the onset of AD [198]. In the early disease stage prior to plaque formation, microglia engage in protective functions such as clearance of Aβ, suppression of tau hyperphosphorylation, and release of neurotrophic factors that prevent symptoms of AD [197,199,200,201,202]. However, as disease progresses, microglial responses are altered and sustained microglia activation contributes to neurodegeneration [203]. This suggests a bimodal microglia activation response throughout the progression of AD, where moderate activation leads to a protective phenotype that is prominent in the preclinical stages, while excessive activation leads to a neurotoxic phenotype that emerges in the clinical phase [198,203]. Therefore, microglia-mediated therapeutic approaches now focus on targeting the initial biochemical event before disease onset, such as treating patients with mild cognitive impairment who have not yet manifested symptoms of dementia.

#### 4.2.3. Microglia Response to Amyloid-β

In the brain, the balance between Aβ production and clearance controls amyloid burden, and mechanisms that regulate Aβ metabolism have been linked to the etiology of AD [204]. Microglia play a key role in Aβ metabolism and clearance, and the cholesterol metabolism pathways involved in phagocytosis or enzymatic breakdown of Aβ are enriched in microglia [205]. In fact, one of the well-known beneficial functions of microglia in the context of AD is their ability to limit plaque formation by clearing pathological Aβ [199].

Aβ modulates various microglial responses, such as chemoattraction, activation, and proliferation, and these cellular responses serve as a protective mechanism to facilitate the first-line defense response and limit further Aβ deposition [206]. Aβ phagocytosis/endocytosis in microglia is mediated by the interaction of Aβ with various microglial receptors, including TREM2, TLRs, CD36, class A1 scavenger receptors (SR-A1) and receptor for advanced glycation end products (RAGE) [207]. The overall response of microglia depends on the specific type of receptor activated and the structural form of Aβ [206].

Various inflammatory pathways are triggered by Aβ and lead to the production of pro-inflammatory cytokines and reactive oxygen and nitrogen species (ROS/RNS). Aβ-mediated activation of the CD36/TLR4/TLR6 complex is largely involved in amplifying pro-inflammatory microglial responses [208], and activation of RAGE also mediates inflammatory responses in microglia and promotes oxidative stress in neurons [209]. Aβ also activates the NLRP3 inflammasome, an intracellular tricomplex containing a sensor protein (NLRP3), an adaptor protein (ASC) and an effector protein (caspase-1), which promotes the production of IL-1β and induces neurotoxicity [210]. NLRP3 inflammasome activation can alter the microglial phagocytosis response and promote Aβ deposition [211], and the release of ASC from activated microglia also facilitates plaque formation by inducing the Aβ oligomerization and aggregation [212]. Transient receptor potential melastatin 2 (TRPM2), a Ca^2+^-permeable non-selective cation channel, has recently been implicated in Aβ-induced AD pathologies [213,214]. In a transgenic AD model, loss of this channel restores Aβ-induced synaptic loss, memory impairment, and microglial activation [214]. Supporting in vitro studies also demonstrated that the TRPM2 channel mediates Aβ-induced microglia activation and TNFα production. This receptor is upregulated by a high level of ROS, and Ca^2+^ influx through this channel was shown to mediate ROS-induced activation of NLRP3 in Aβ-activated microglia [215]. Overall, Aβ can activate various inflammatory and neurotoxic pathways in microglia that can mediate AD pathology.

Microglial senescence upon aging can sensitize microglia to inflammatory signals and exacerbates Aβ-pathology [216]. While Aβ can trigger chronic activation of microglia, inflammation in the brain microenvironment can, in turn, weaken clearance mechanisms and promote Aβ deposition, suggesting a positive feedback loop for AD pathology. For example, pro-inflammatory cytokines produced by microglia such as TNFα and IFNγ can diminish the ability of microglia to degrade Aβ and suppress the expression of Aβ-degrading proteases, contributing to plaque formation [217,218]. Pro-inflammatory cytokines also upregulate microglial iNOS in the AD brain, leading to toxic NO production [219,220]. High concentrations of NO in the brain can alter mitochondrial function, induce neurotoxicity, and trigger nitration of Aβ particles, which promotes plaque aggregation [221]. Furthermore, other microglial activation DAMPs, such as chromogranin A and myeloid-related protein 14 (MRP14), can trigger chronic neuroinflammation in the AD brain and further compromise the microglial phagocytosis response, thus contributing to plaque build-up [222,223].

Evidence suggests that Aβ accumulation in the early phase of AD is caused, in part, by impaired microglial clearance mechanisms. For example, a dysfunctional autophagic response is evident in the AD brain and has been attributed to a reduced expression of microglial beclin-1 [224], a protein that plays an important role in Aβ phagocytosis by promoting the recycling of scavenger receptors such as CD36 and TREM2 [225]. Aβ deposition and buildup of senile plaques increase with age and may be related to age-associated microglial senescence, which impairs the ability of microglia to detect phagocytic targets [216]. In support of this, various microglial Aβ-binding cognate receptors involved in the uptake of Aβ, such as CD36, SR-A, and RAGE, are reduced in aged mice and AD brain [226], suggesting an impaired phagocytosis response with age. In addition, aging studies comparing isolated microglia cultured for 2 or 16 days showed that older microglia have a lower phagocytosis and autophagic response after Aβ treatment, accompanied by reduced expression of TREM2 and an increased expression of the senescence-associated β-galactosidase (SA-B-gal) [227]. Furthermore, a study using an ex vivo organotypic coculture model demonstrated that young microglia could restore the amyloid plaque clearance capacity of aged microglia [228]. These findings indicate that dysfunctional microglial Aβ clearance contributes to plaque build-up upon aging, thus targeting age-associated microglial senescence has therapeutic potential to prevent the onset and progression of AD. Alternatively, replacing older microglia with young functional phenotypes that express sufficient levels of autophagic receptors may be a promising candidate for novel cell transplantation therapy for AD. This type of microglia replacement approach has recently been attempted in a different model of ND using cutting-edge tools that incorporate bone marrow and microglia-depleting chemicals [229].

#### 4.2.4. Microglia Response to Tau

Tau is a microtubule-associated protein that is abundantly expressed in neurons and is normally involved in axonal microtube assembly and stabilization [230]. Post-translational modifications play an important role in modulating tau functions; in particular, hyperphosphorylation of tau impairs the interaction of tau with microtubules and induces abnormal folding that promote aggregation and the formation of neurofibrillary tangles (NFTs) [230]. Activated microglia are observed in close proximity to NFTs in AD patients [231], and compelling experimental evidence demonstrated both a beneficial and detrimental role of microglia in tau pathology. Microglia can engulf and degrade pathological tau particles, but can also propagate the spread of tau pathology [232,233].

In experimental AD models, activated microglia populations are evident prior to tau deposition [234,235], thus implicating the role of microglial inflammation in exacerbating tau aggregation and spreading. In support of this, known immunosuppressants that modulate microglia activity have been used to treat AD and have shown attenuation of tau pathology [236,237]. Microglia can contribute to the spread of tau pathology through various mechanisms. Studies have shown the presence of hyperphosphorylated tau in aged dystrophic microglia [238], suggesting that impairment of clearance mechanisms upon aging can lead to intracellular accumulation of pathological tau particles that promote microglia dystrophy. Impaired processing of pathological tau in microglia can contribute to tau propagation by releasing tau-containing exosomes/microvesicles (MV) [233]. High concentrations of MVs containing bioactive molecules are evident in the AD brain, and the release of bioactive mediators from these MVs, including toxic tau seeds, is known to alter the brain microenvironment and promote microgliosis and tau spreading [239,240].

Studies have shown that NFT formation is accelerated by microglial inflammation [233,241]. Microglial uptake of tau can also trigger activation of the NLRP3 inflammasome complex, which promotes tau seeding [210]. Activated microglia can also modulate tau function through post-translational modifications. For example, pro-inflammatory cytokines released by activated microglia, such as IL-1 and IL-6, induce tau phosphorylation, thus promoting NFT formation [242]. Microglia also promote tau ubiquitination, and ubiquitinated forms of tau are better incorporated into exosomes that can facilitate extracellular release of tau seeds [233]. Pro-inflammatory responses in microglia can also disrupt regulatory CX3CR1-CX3CL1 signaling [243], which can further lead to sustained microglial activation and promote NFT formation [18].

Proteasomal and autophagic mechanisms are involved in tau degradation, and there is evidence that these clearance mechanisms are impaired in AD [244]. The receptors involved in tau interaction are not well characterized. However, Bolos et al. (2017) demonstrated that the microglial CX3CR1 receptor interacts with tau and promotes its phagocytosis and internalization, and S396 tau mutant with impaired binding to CX3CR1 is associated with a defect in tau clearance [245]. However, in the AD brain, tau competes with CX3CL1, and sustained CX3CR1/tau signaling can amplify pro-inflammatory and neurotoxic pro-inflammatory responses [246]. The exact mechanism for the dual role of microglia is not clear; however, microglia probably mediate different effects on tau pathology, depending on the stage of the disease. Overall, microglia-induced tau pathology is facilitated by a combination of factors that involve a defect in tau clearance mechanisms and neuroinflammatory mechanisms that promote tau aggregation and propagation. More studies are needed to characterize the role and mechanism of the reciprocal microglia-tau interaction at different stages of AD progression.

#### 4.2.5. Microglia Morphologies and Phenotypes in AD

Various immunohistology studies in transgenic AD models and human clinical samples demonstrated the presence of various morphologically distinct populations of microglia in AD brain, including traditional ramified resting microglia and amoeboid-shaped activated microglia [247]. Amoeboid-shaped microglia are predominantly localized in the hippocampus and cerebral cortex region where amyloid plaques and NFTs are commonly found, while ramified microglia are dispersed away from these pathogenic regions [247,248,249]. A dystrophic/degenerating microglia phenotype, exhibiting thin fragmented processes, is also reported in the hippocampus of human AD autopsy brains [41].

Some of the activated microglia population located near senile plaques assume an elongated and polarized morphology, commonly referred to as bipolar/rod-shaped microglia, and these microglia phenotypes are mostly aligned end-to-end in the CA1 and CA2/3 region of the hippocampus [41,250]. Bipolar/rod-shaped microglia are evident in the affected brain region in the early phase of AD, and as disease progresses, amoeboid microglia predominate, while bipolar microglia appear in less affected brain regions in the later stage of AD [37,40,41]. Compared to amoeboid microglia, in vitro studies demonstrated that bipolar/rod-shaped microglia highly express low levels of pro-inflammatory markers and can transform into an amoeboid phenotype by LPS treatment [38,251]. Transformation towards an amoeboid phenotype enhanced the production of proteolytic extracellular matrix-degrading molecules, suggesting a neurotoxic role of amoeboid microglia [252]. Although these observations implicated a potential neuroprotective effect of bipolar/rod-shaped microglia in AD pathology, more studies are needed to understand the exact role and regulation of this microglia phenotype.

A recent ultrastructural analysis using high-resolution transmission electron microscopy (TEM) revealed a unique phenotype of microglia known as ‘dark microglia’ (DM), which is named for its characteristic dark appearance under TEM [253,254]. This phenotype, which was nearly absent in normal adult brains, was abundant under conditions of chronic stress, aging, and in a transgenic AD model. DM exhibited a distinct ultrastructural morphology and showed signs of severe oxidative stress, such as condensed cytoplasm and nucleoplasm, abnormal nuclear chromatin, dilated ER and Golgi body, and mitochondrial alterations. Furthermore, this microglia phenotype is highly reactive and rich in endosomes, implicating a strong phagocytic function. Unlike normal microglia with shorter and thicker processes, DM have thin and highly branched ramifications that enable them to extensively circle and engulf axon terminals, dendrites, and entire synapses [253,254].

DM strongly express the CD11b microglia receptor while encircling synaptic elements and have high expression of TREM2 when associated with amyloid plaques; these receptors have important synaptic pruning and phagocytosis functions, suggesting an important function of DM in pathological remodeling of synapses [253,254]. The significance of DM has not yet been elucidated, but its distinct structural properties, specific occurrence in aging and chronic stress environments, and its implicated role in pathological synapse remodeling suggest an important function of DM in AD pathology. DM has recently been identified in humans and other species [255,256], suggesting that this microglia morphology is highly conserved across species and may modulate disease progression (for detailed reviews on DM, refer to [253,257]). Overall, the presence of various microglia morphologies demonstrates a complex role of microglia in AD pathology.

#### 4.2.6. M1 and M2 Microglia in AD

The dichotomous paradigm of M1 and M2 is less commonly applied in the context of AD; however, it is sometimes used as a general reference to describe either pro-inflammatory microglia or a phagocytic phenotype, which is distinguished based on molecular markers and the overall functional response. It is believed that M2 microglia are efficient phagocytes and mediate protective functions, while M1-like pro-inflammatory microglia have a poor ability to clear Aβ and toxic tau, thus exacerbating AD pathology [258]. Inhibition of pro-inflammatory responses, such as inhibition of the NLRP3 inflammasome, has a protective effect in reducing amyloid burden and associated toxicity [259]. Pro-inflammatory cytokines released by M1-like microglia, such as IFNγ and TNFα, are implicated in AD pathology, as they can inhibit the uptake of Aβ or the degradation of internalized Aβ [217,218]. In addition to their role in Aβ pathology, pro-inflammatory microglia can also mediate tau neurotoxicity by triggering tau hyperphosphorylation [241]. In fact, the presence of pro-inflammatory microglia has been reported in vivo in transgenic AD models before the manifestation of tau neurotoxicity [234], suggesting a detrimental role of M1 microglia in tau pathology.

Studies have shown that microglia surrounding amyloid plaque exhibit an activation profile of M2-like microglia, and this microglia phenotype is largely associated with protective functions [198,203,260]. Both in vitro and in vivo findings demonstrated that microglial polarization towards an M2 phenotype alleviates neuroinflammation, M1-mediated neurotoxicity, and AD pathogenesis [261]. M2 polarization reduces microglial reactivity towards pathogenic forms of Aβ, which helps limit Aβ-toxicity [47]. Unlike the pro-inflammatory M1 phenotype with impaired phagocytosis function, M2-like microglia exhibited enhanced Aβ phagocytosis and clearance [258], which implies a beneficial role for the M2 phenotype in AD pathology. M2 microglial responses can be diverse, depending on the specific M2 subtype. For example, the M2a phenotype, which is induced by IL-4, has enhanced phagosome/lysosome function and increased scavenging capacity, which facilitates Aβ degradation [262,263], while the M2c phenotype is involved in tissue repair and wound healing functions [258]. In general, chronic induction of the M1 pro-inflammatory phenotype or defect in M2-like phagocytic and wound-healing microglia can exacerbate AD pathogenesis.

Studies exploring the role of cytokines in AD pathology have yielded mixed findings. In the APP transgenic model, where memory deficit and amyloid plaque manifest at 3 and 6 months, respectively, intracerebral administration of IL-4 and IL-13 was shown to have neuroprotective effects in limiting plaque build-up and improving cognitive functions of mice at 4.5 and 9 months [264]. However, in a different mouse model with preexisting amyloid plaques, adenovirus-based overexpression of recombinant IL-4 at 4 months, while inducing M2 polarization, exacerbated amyloid deposition after 6 weeks [265], suggesting that IL-4 impairs microglial phagocytosis response. Although these studies did not use the same model, the discrepancies in their findings may suggest that individual cytokine treatments may not be sufficient to improve AD recovery, or that the therapeutic impact of anti-inflammatory cytokines such as IL-4 may be limited to a specific time frame during disease progression. A paradoxical beneficial role of pro-inflammatory cytokines (IL-6, IFNγ, and TNFα) has also been reported, such as enhancing complement-mediated Aβ phagocytosis and clearance [266,267,268]. These findings suggest diverse roles of cytokines and microglia phenotypes in AD pathology, which can vary depending on the specific disease stage. For example, it is suggested that Aβ clearance in AD may be primed by the M1-like microglia response and maintained by M2 microglia [47,242]. Although M1 microglia have a potential role in Aβ uptake, sustained M1 polarization and chronic inflammation in the AD brain can overwhelm the functional response of M2 microglia and lead to impaired Aβ clearance and M1-neurotoxicity.

#### 4.2.7. Timeline of Microglia Phenotypes in AD

Studies have explored age-associated changes of microglia phenotypes in the context of AD. Jimenez et al. (2008) characterized age-dependent alteration of microglia phenotypes in the hippocampus of double PS1xAPP transgenic AD mice [260]. They observed that YM1-positive M2 microglia with low expression of pro-inflammatory markers predominated at 6 months, and later switched to M1-like microglia at 18 months. The M2-phenotype microglia in 6-month-old mice were exclusively located surrounding Aβ plaque and exhibited phagocytic abilities, while the M1 activation response in the older age group was more widespread and included plaque-free brain regions. This M2-to-M1 switch also coincided with significant neurodegeneration and accumulation of Aβ oligomers, further suggesting the loss of microglial phagocytosis response. This age-dependent impairment of Aβ clearance has also been demonstrated in vitro in aging microglia [227]. Overall, these findings imply that microglia transition from a phagocytic and protective phenotype, in the early stage of Aβ pathology, to a pro-inflammatory and neurotoxic phenotype in advanced stages.

Longitudinal PET imaging studies in patients with MCI and AD have also revealed a similar microglia activation profile in human subjects where a protective phenotype is believed to be present in the early stage of AD, while a pro-inflammatory phenotype is prominent as the disease progresses and Aβ clearance fails [198,203,269]. M2-like microglia peak in the MCI stage of disease while the M1 phenotype is more apparent in symptomatic AD patients [269]. By the time symptoms manifest, protective microglia have already transitioned to a dysfunctional phenotype [203], signifying a narrow therapeutic window to preserve the functional response of protective microglia.

The transition from M2-to-M1 phenotype is attributed to a change in the brain microenvironment. In the early presymptomatic phase of AD, there is moderate activation of microglia and protective functions of microglia dominate, such as clearance of Aβ/tau. On the contrary, a sustained inflammatory environment during AD progression can alter microglial housekeeping and sensing functions, as well as the host defense mechanism, thereby promoting neuroinflammation and neurotoxicity [270]. For example, there is evident dysregulation of endogenous microglia regulatory molecules such as loss of CX3CR1 and TREM2 that can cause microgliosis and exacerbate AD pathology [241,271] While these findings implicate a dynamic microglia activation profile, the overall response of microglia in AD is more complicated than what is described by the two distinct activation phenotypes.

Colton and colleagues (2006) described a complex pattern of microglial gene expression in AD patients and two different transgenic mouse models: the APPsw model for amyloid deposition and the Tg-SwDI model for cerebral amyloid angiopathy [272]. Assessment of different phenotypic markers showed a heterogeneous gene expression profile in the AD brain representing a mixture of classical and alternative activated states. Microglia response in AD can be diverse due to different activation triggers, e.g., Aβ vs. tau. Amyloid plaques and NFTs also have regional distributions that can lead to a heterogeneous activation response of microglia in the diseased brain [273]. Furthermore, extracellularly deposited mediators such as Aβ can activate the peripheral immune response and elicit infiltration of other immune cells into the brain, which can modulate microglia phenotypes [274]. While these findings highlight the complex activation response of microglia, a specific phenotype of microglia may dominate at different stages of AD. Therefore, longitudinal studies characterizing stage-specific responses of microglia in different regions of the brain can help identify an optimal therapeutic time window to specifically target detrimental microglia phenotypes.

#### 4.2.8. Diversity of Microglia in AD Inferred from Transcriptome Studies: DAM and HAM

Transcriptome studies that examine microglial signatures in AD models have highlighted the complex nature of the microglial response in AD pathology [98,275,276]. Orre et al. (2014) reported transcriptome alterations observed in a late-stage (15–18 months) APP/PS1 transgenic mouse model and revealed that microglia in AD brain exhibit lower expression of genes important for phagocytosis and endocytosis functions, while showing upregulation of innate activation genes, such as those involved in antigen processing and presentation responses [98]. Wang et al. (2015) identified similar remodeling of the microglial transcriptome in 8.5-month-old plaque–depositing AD model (5XFAD, harboring five AD-linked mutations) that represented a late stage of AD progression [275]. Using coexpression network analysis, Holtman et al. (2015) compared the microglial transcriptome in mouse models of aging, AD, and ALS and identified shared transcriptional profiles such as dysregulation of genes involved in antigen presentation, AD signaling, and phagocytosis [85]. However, they observed a highly heterogeneous microglia transcriptome profile, which varied by age and brain region. Recently, Lopes et al. (2022) identified similar regional and age-related differences in the microglia transcriptome of human AD brain tissue, including alteration in a wide range of inflammatory responses such as the IFN, glucocorticoid, STAT3, and IL-6 signaling pathways [95].

Transcriptome analyses have also revealed a novel disease-associated microglia (DAM) phenotype found near Aβ plaques [277,278]. Krasemann et al. (2017) identified a novel molecular signature associated with the induction of DAM phenotype; the switch to DAM was triggered by phagocytosis of apoptotic neurons and depended on the activation of TREM2-APOE signaling and subsequent suppression of homeostatic microglia signatures [277]. In a plaque-deposited mouse 5XFAD model, Keren-Shaul et al. (2017) also identified a subset of protective DAM population enriched near senile plaques that exhibited a distinct molecular signature compared to microglia in the normal brain [278]. In the same disease model, Grubman et al. (2021) transcriptionally profiled amyloid plaque containing microglia (labeled with methoxy-XO4, XO4^+^) and plaque-free microglia (XO4^−^) during disease progression and showed that XO4^+^ microglia were functionally distinct, as defined by active fibrillar Aβ phagocytosis [279]. Their study also identified distinct processes such as accelerated aging and direct response to plaque phagocytosis that were associated with microglial changes in AD.

DAM activation occurs through two sequential steps: The first activation step is considered an intermediate state and involves TREM2-independent downregulation of homeostatic markers such as P2RY12, while the second activation state is TREM2-dependent and is associated with overexpression of genes involved in lipid metabolism and phagocytosis functions [278]. In vitro study showed that age-associated reduction in TREM2 in older primary microglia culture correlates with impaired autophagic response after Aβ treatment compared to microglia cultured for a short period of time [227]. Therefore, TREM2-dependent activation of DAM is thought to play a protective role in AD pathology. DAM-like phenotype was also identified in AD patients and in other diseases such as ALS [280]. So, DAMs may exist in various NDs that exhibit a characteristic defect in clearance of pathogenic proteins, and this microglia phenotype may mitigate disease progression through phagocytosis of misfolded and aggregated proteins.

Using single-cell RNA sequencing (scRNA-seq) technology, Mathys et al. (2019) profiled ~80,000 single-nucleus cortical transcriptome samples from 48 patients with varying degrees of AD pathology and identified transcriptional profiles linked to AD pathogenesis, such as altered expression of genes involved in inflammation and Aβ clearance pathways, including APOE, TREM2, MHCII, and C1qb [281]. Age-related dysfunction of the microglial endolysosomal system has also been evident in human bulk RNA-seq samples from AD patients, such as alteration of the GTPase-activating protein USP6NL and the phosphatidylinositol-binding clathrin assembly protein (PICALM), which function to regulate Aβ endocytosis and processing [95].

While the aforementioned transcriptome studies were performed in RNA samples isolated from postmortem brain tissue, Olah et al. (2020) recently performed scRNA-seq experiments in live microglia cells purified from human cerebral cortex and identified 9 diverse clusters of human microglia subpopulations enriched for disease-related genes [282]. A specific antigen-presenting cluster was histologically verified and appeared to be prominently enriched for AD genes whose expression was suppressed in AD patients. A significant portion of the genes identified in the different clusters of human microglia appeared to overlap with signature genes previously reported in murine DAM phenotypes [278]. A recent study by Srinivasan et al. (2020) also characterized a human Alzheimer’s microglia (HAM) profile from frozen postmortem brain tissue using bulk RNA-seq and identified similar profiles between DAM and HAM genes, particularly in AD risk factor genes involved in lipid transport and lysosome biology such as APOE, CLU, PLCG2 [283]. However, a detailed comparison of their data with a recently published human microglia scRNA-seq and snRNA-seq datasets [281,284,285], revealed a distinct profile of HAM microglia, such as upregulated expression of certain HAM genes that do not change (PTPRG and IL15) or are not expressed (GYPC and DPYD) in DAM. The DAM phenotype is evident in other NDs, such as multiple sclerosis, and is mainly characterized in rodent AD models with implicated protective roles in Aβ pathology [278]. However, HAM showed an enhanced human aging phenotype and a unique transcriptome profile specific to human AD and exhibit defective activation and functional responses [286]. Aside from species-specific intrinsic differences, the distinction between DAM and HAM profile can be attributed to experimental factors; many transgenic AD models harboring Aβ-related mutations mimic Aβ pathology, which is an early stage of AD before the manifestation of neurodegenerative symptoms [287]. Therefore, microglia, responses in rodent models may not represent features of clinical AD. While advanced genome-wide analyses have revealed the complex and heterogeneous nature of microglia, additional longitudinal transcriptome and functional studies in various age groups are necessary to better understand the stage-specific response of microglia in human AD. Furthermore, future single-cell RNA-seq studies coupling functional and histological data are needed to categorize the transcriptionally identified microglia clusters as different microglia phenotypes (refer to Stratoulias et al., 2019 [288] for further details on microglia diversity and subtype categorization in health and disease).

### 4.3. Microglia in Parkinson’s Disease

#### 4.3.1. Parkinson’s Disease (PD)

Parkinson’s disease (PD) is the second most prevalent neurodegenerative disease and is characterized by a progressive and selective loss of midbrain dopaminergic (DA) neurons in the substantia nigra pars compacta (SNpc) and nerve terminals in the striatum [289]. This leads to gradual impairment of DA transmission in the motor regions of the striatum that causes dysfunctional motor functions such as bradykinesia, tremor, and rigidity [289]. Although PD is mainly considered a movement disorder, motor deficit usually occurs at an advanced stage after approximately 50% of DA neurons are lost [290], and patients experience many nonmotor symptoms in the early phases, such as cognitive impairment, autonomic dysfunction, and psychiatric changes [289]. The main pathological hallmark of PD is the formation of Lewy bodies (LBs), which are abnormal intracellular inclusions containing aggregated proteins largely made up of α-synuclein (α-syn) [291].

Aging is the major risk factor for PD, but the exact mechanism for the onset of PD is unknown and likely involves a combination of factors such as genetic and environmental predisposition [292]. PD can either be familial (fPD), which is a less common form primary caused by genetic factors, or sporadic (sPD) which has a multifactorial origin and is a more prevalent form. More than 30 loci are identified as risk factors for PD, some of which are extensively studied, such as mutations in genes encoding synuclein (SNCA), leucine-rich repeat kinase 2 (LRRK2), parkin (PRKN), and Parkinson disease protein 7 (PARK7) [293]. In addition to genetic susceptibility, sedentary lifestyle, poor diet, and exposure to environmental toxins (eg herbicide/pesticides) also contribute to a lifetime risk of developing PD [292].

Various pathogenetic mechanisms contribute to PD-mediated neurotoxicity, including impairment in α-syn proteostasis, mitochondrial dysfunction, oxidative stress, glutamate excitotoxicity, and neuroinflammation [292,293]. Developing effective therapies for PD has been a challenge due to the complex etiology and mechanism of PD and the diverse neuropathological changes and symptoms among patients. Therapeutic options for PD are mainly limited to symptomatic interventions focused on dopaminergic pharmacological targets, such as the FDA-approved dopamine replacement agent levodopa (l-DOPA), a dopamine precursor used to restore decreased dopamine levels in patients [289]. Although this treatment option relieves motor symptoms, it does not target the underlying mechanism of neurodegeneration and is not effective in slowing the progression of PD [294]. A better understanding of the underlying mechanism of PD pathogenesis and the identification of early biomarkers is crucial for the development of preventive and neurorestorative therapies.

One of the most prevalent pathogenic players in PD is α-syn. This 140 amino acid protein is highly expressed in the brain and its aggregated toxic form constitutes the core of LBs [295,296]. Monomeric and aggregated forms of α-syn are commonly found in the brain, CSF, and blood of PD patients [297,298]. α-syn aggregation in the brain is a characteristic feature of both sPD and fPD, and various pathogenic mechanisms contribute to aggregation, including changes in the SNCA gene (point mutations, duplications, and triplication) and various post-translational modifications of the protein (e.g., phosphorylation, oxidation, nitration, ubiquitination) [296]. Therefore, preclinical studies in PD commonly use rodent models with treatment or overexpression of human α-syn, or transgenic models harboring α-syn mutations. α-syn homeostasis in the CNS is regulated by the proteasomal and autophagic degradation systems [299], and age-related progressive decline in these α-syn clearance mechanisms contributes to α-syn accumulation in the aged brain [300,301]. In addition to age-associated changes, various genetic mutations linked with fPD, such as the G2019S mutation in LRRK2, are associated with decreased a-syn homeostasis [302]. Furthermore, alterations in the brain microenvironment, including oxidative stress and inflammation, also play an important role in the pathogenic mechanism of α-syn [303].

#### 4.3.2. Microglia Activation in PD

Collective evidence from human postmortem analysis, in vivo PET imaging, and gene-wide association studies (GWAS) has revealed genetic and neuropathological evidence for the contribution of microglia in PD pathology [57]. The nigrostriatal system, particularly the SN region, has a high abundance of microglia, and neurons within this region are very sensitive to inflammatory and oxidative damage [304]. An early study by McGeer et al. (1988) provided the first indication of microgliosis in the SN of postmortem PD tissue [305]. Langston et al. (1999) further revealed the presence of chronic microgliosis in the postmortem human brain exposed to MPTP [306], a neurotoxin that triggers acute and irreversible human parkinsonism [307]. Interestingly, activated microglia were present even up to 16 years after intoxication, suggesting a sustained inflammatory response. Additionally, in vivo imaging employing inflammatory and microglia activation markers revealed that activated microglia are present in early stage PD, and sustained activation and inflammatory response in the brain are positively correlated with disease progression [308,309,310,311]. Activation of microglia was also observed in the midbrain region of patients with REM sleep disorder, which is considered the prodromal phase of PD [312]. Unlike the region-specific activation response shown in the early stage, a more widespread microglial activation response was evident in additional regions of the brain, including the hippocampus and cortex of diagnosed patients [313,314]. GWAS further showed that key PD-susceptibility genes encode microglial proteins that play important roles in immune regulation and microglial phagocytosis, such as LRRK2 [315]). Overall, these pieces of evidence suggest that microglia play an essential role in the pathogenesis of PD and that their activation response is highly dynamic, exhibiting regional and stage-specific heterogeneity.

#### 4.3.3. Microglia Response to α-Synuclein

A key neurotoxic mechanism of α-syn in PD is the activation of microglia to promote neuroinflammation [316]. In the PD brain, activated microglia are commonly associated with α-syn-containing LBs [317], and α-syn released from degenerating DA neurons is an endogenous DAMP that activates microglia [316]). In vivo studies in transgenic and neurotoxin-based PD model demonstrated that activated microglia and inflammatory responses are evident and precede neurotoxicity [318,319]. This suggests that neuronal death is not a necessary event for microglial activation and that microglial inflammatory responses likely mediate α-syn neurotoxicity.

Reciprocally, a key neurotoxic mechanism of activated microglia is their ability to facilitate α-syn misfolding and propagation, which can subsequently cause microgliosis and exacerbate disease progression [320]. For example, α-syn can activate NADPH oxidase (Phox) and trigger the release of ROS from microglia, creating an environment of oxidative stress [321]. This response can further induce α-syn oxidation in neighboring neurons and promote α-syn aggregation, propagation, and disease progression [322]. In turn, modified α-syn (e.g., mutated, fibrillar, and oligomeric forms) has a stronger propensity to activate microglia and cause more microgliosis and neurodegeneration [323]. Specifically, misfolded α-syn can trigger a reactive M1-like pro-inflammatory phenotype. For example, a specific mutant form of α-syn (A53T), which is linked to fPD, induces stronger activation of the NFκB/AP-1/Nrf2 pathway that leads to the production of pro-inflammatory cytokines and ROS, compared to wild-type α-syn [324]. Furthermore, oligomeric/fibrillar α-syn activates microglial NLRP3 inflammasome, which can lead to the release of IL-1β and ASC in the extracellular space, promoting a pro-inflammatory response [325]. In general, these findings suggest a positive feedback loop in which α-syn disorder and microglial neuroinflammatory processes propagate one another and drive chronic neurodegeneration.

Microglia activation in response to α-syn also mediates protective effects; it triggers a defense response that limits disease progression by modulating α-syn clearance and toxicity [323]. Microglia are capable of internalizing and degrading α-syn, and studies have shown that ingested α-syn is sequestered by the microglial autophagosome complex for degradation [326]. Microglia-mediated clearance of α-syn is regulated by LRRK2, and a pathogenic variant of LRRK2, which is evident in patients with PD, affects the ability of microglia to internalize and degrade α-syn thereby contributing to PD [315]. However, the protective function of microglia in α-syn clearance is weakened during aging and after sustained activation of microglia, and defects in microglial phagocytosis mechanisms can contribute to α-syn accumulation, microgliosis, and neurotoxic response [301,327]. Bido et al. (2021) recently used a novel mouse model with selective overexpression of α-syn in microglia and demonstrated that these mice, albeit lacking endogenous aggregated α-syn, develop progressive DA neurodegeneration by phagocytic exhaustion and oxidative stress [328], further supporting the notion that α-syn responses in microglia can drive neurotoxicity. The vicious cycle of phagocytic defect and oxidative/pro-inflammatory toxicity in α-syn-accumulating microglia leads to progressive DA damage. However, microglial α-syn also plays an important for homeostatic lipid signaling [323], and its loss has been shown to impair phagocytosis and promote excess COX-2 activation in response to inflammation [329]. Therefore, microglial response to α-syn is complex and varies depending on the α-syn forms, and the solubility and aggregation properties of α-syn change during disease progression, which affects the overall response of microglia [330].

Microglial uptake of α-syn and subsequent activation are mediated by mechanisms and receptors similar to those involved in Aβ-signaling, prominently TLR2, TLR4, and the scavenger receptor CD36 [270]. Activated microglia expressing TLR2 are present at the site of neurodegeneration in patients with PD [313], and α-syn interaction with this receptor mediates pro-inflammatory and neurotoxic responses [316]. The expression of TLR2 in SN is higher in PD patients, predominantly in the prodromal stage, suggesting a detrimental role of this receptor in early disease processes [313]. CD36 activation facilitates uptake of α-syn and microglial activation, and by forming a complex with TLRs, it induces pro-inflammatory responses [208,316]. CD36 and TLR2 are involved primarily in the uptake of oligomeric or fibrillar α-syn [323,331], and their harmful role in PD may be attributed to their sensitivity to toxic forms of α-syn.

TLR4-mediated α-syn uptake in microglia is not limited to aggregated forms of α-syn, and activation of this receptor has been linked to protective functions such as enhanced endocytosis response and autophagosome function [323]. In vitro and in vivo studies by Choi et al. (2020) demonstrated that α-syn interaction with TLR4 and the subsequent activation of NFκB induces transcriptional upregulation of the autophagy receptor SQSTM1/p62, which is involved in autophagic clearance of α-syn inclusions [326]. However, activation of TLR4/NFκB pathway can also promote pro-inflammatory responses and ROS release, which contributes to neurodegeneration. Given that TLRs have temporal and regional activation in PD models [332], the precise role of TLR4 in PD pathology may depend on the timing of activation and the severity of the disease.

In addition to direct interaction and activation of microglia receptors, α-syn can also activate microglia through other indirect mechanisms involving the release of microglial mediators. For example, α-syn induces the release of MMPs that subsequently activate microglial PAR-1 receptors [333]. Furthermore, while the microglial response to PD is extensively studied in the context of α-syn toxicity, microglia can also be activated by other mediators to promote pro-inflammatory responses. In fact, microgliosis occurs in various experimental PD models that utilize neurotoxins such as MPTP, 6-OHDA, and the organic insecticide rotenone. Some of the mediators that activate microglia in PD include dopamine-derived neuromelanin and mitochondrial DAMPs such as mitochondrial DNA (mtDNA), cytochrome c, and ROS [37,334]. Overall, the progression of PD is exacerbated by a self-propagating cycle of microglial activation and neurotoxicity, driven by a combination of factors that include neuroinflammation, α-syn aggregation, oxidative stress, and mitochondrial and autophagy dysfunction.

#### 4.3.4. Microglia Phenotypes in PD

##### M1 and M2 Microglia in PD

Collective evidence from postmortem human brain analysis and in vivo studies suggests that the M1-like pro-inflammatory phenotype of microglia is prominent in PD. Specifically, M1 microglia markers are elevated in PD clinical samples and largely cluster near α-syn deposits in the SN of patients [314,335]. Furthermore, most activated microglia observed in various experimental PD models, utilizing either α-syn, environmental toxins and inflammatory agents, exhibit a characteristic M1-like activation response [47]. Some of the key genetic variants of PD identified in GWAS studies, such as LRRK2, parkin, and DJ-1, also involve M1-like pro-inflammatory responses [47,57]. Studies have also demonstrated a positive correlation between pro-inflammatory microglial responses and aging, as well as DA neuronal loss [75,336], suggesting that therapeutic targeting of the pro-inflammatory microglia phenotype can help protect vulnerable midbrain DA neurons from inflammation-induced neurodegeneration. In support of this, strong microglial COX-2 and iNOS reactivity is observed in PD patients [337,338], and COX-2 activity in neurotoxin model of PD suppresses microglial activation and secondary DA neurodegeneration [339]. Furthermore, studies in a transgenic PD model demonstrated that microglial activation and the production of pro-inflammatory cytokines such as TNFα, IFNγ, and IL-1β precedes neurotoxicity [318,319], and inhibition of these cytokines suppresses microglia-mediated neurodegeneration [57]. Overall, these findings strongly suggest that M1-like microglial responses contribute to neurodegeneration in PD.

The role of M2 microglia in PD pathology is not well understood, and limited studies have linked M2 microglia in α-syn toxicity and PD. Glatiramer acetate (GA) is an FDA-approved drug for multiple sclerosis and promotes anti-inflammatory M2 activation [340]. In experimental PD model, GA treatment reversed MPTP-induced motor dysfunction and restored striatal dopamine levels [341], implicating a potential neuroprotective role of M2 activation in PD pathology. In both LPS-inflammation and MPTP-model of PD, human IL-10 infusion limited microglial activation and protected against DA neuronal loss in the SN [342,343]. Although these results suggest a potential anti-inflammatory and neuroprotective role for this cytokine, a detrimental effect of IL-10 has also been reported. In a recent study, Cockey et al. (2021) used AAV to express recombinant IL-10 and its immunosuppressive variant I87A (vIL-10) in a mouse model of synucleinopathy and showed that sustained intraspinal expression of both IL-10 forms resulted in shorter lifespan, associated with increased microgliosis, neuronal autophagy dysfunction, accelerated α-syn pathology, and increased apoptosis [344]. Various contradictory studies have also assessed IL-10 expression in human PD brains. A high circulating level of IL-10 is detected in patients with PD patients and was shown to correlate with various nonmotor symptoms of PD, such as anxiety, depression, and gastrointestinal dysfunction [345,346], suggesting that this anti-inflammatory cytokine is involved in the pathogenic mechanism of PD. However, there is no conclusive consensus on the correlation between IL-10 and PD, as other reports showed negative correlation between IL-10 and PD-related pain [347].

The anti-inflammatory cytokine IL-4 is also involved in PD pathology. In mixed neuron-glia culture, inhibition of endogenous microglia-derived IL-4 enhanced MPTP-induced neurotoxicity, and exogenous administration of IL-4 in the mouse MPTP model protects against neurotoxicity, indicating a potential protective role of IL-4 in PD [348]. In addition to IL-4 and IL-10, the pleiotropic M2 cytokine TGF-β mediates neuroprotective functions in inflammation and neurotoxin PD models; it limits M1 microglial activation and dopaminergic neurodegeneration [349,350]. Specifically, microglia-derived TGF-β2 is beneficial in PD pathology; addition of exogenous TGF-β2 peptide in TGF-β-depleted microglial conditioned media has been shown to counteract 6-OHDA-induced neurotoxicity of cerebellar granule neurons [351]. Despite its implicated neuroprotective effect, the concentration of TGF-β is elevated in the CSF of PD patients [352], and its role in PD is not clear. Overall, the contradictory findings on the role of M2 cytokines highlight the complex function of anti-inflammatory cytokines in PD pathology.

There is a lack of knowledge on the overall activation trajectory of the M1-like pro-inflammatory and M2-like anti-inflammatory phenotypes during the progression of PD. A study using a human α-syn overexpression model demonstrated a progressive increase in pro-inflammatory M1 markers after 2 and 4 weeks of α-syn induction, while expression levels of M2 markers were not significantly altered [353]. However, this study did not examine the activation profile in an advanced stage with apparent neurodegeneration. Therefore, additional longitudinal studies are needed to understand the dynamic M1/M2 activation profile in PD. Overall, microglial response in PD is heterogeneous and cannot be simplified by the distinction of M1 or M2 markers. There are also morphologically diverse microglia phenotypes in PD, including bipolar/rod-shaped microglia that is also prominent in AD. Bipolar/rod-shaped microglia are present in the SN of PD patients surrounding degenerating DA neurons [305], and in an experimental PD model, this phenotype emerged in the early stage before transitioning to a neurotoxic amoeboid phenotype, suggesting a potentially protective role for bipolar/rod-shaped microglia [354].

##### Microglia Diversity in PD Inferred from Transcriptome Studies

Recent advances in single-cell isolation techniques have enabled the transcriptional profiling of microglia in the PD brain, which has revealed the complex response of microglia. Mastroeni and colleagues (2018) used single-cell laser microscopy technology in conjunction with RNA sequencing to specifically assess microglia-specific transcriptome changes between normal elderly control brains and in AD and PD-diseased brains in two pathologically affected brain regions: the SN (a PD-susceptible region) and the hippocampus region (prominent in AD pathology) [355]. Their findings identified regional and disease-specific heterogeneity in microglial response. Interestingly, despite the prominent role of microglia in inflammatory responses, inflammation-related transcripts did not appear to be the most significantly altered changes in the diseased brain. Aldosterone synthesis, ROS pathways, and biological processes related to behavior were enriched in PD samples, whereas neuronal repair and viral response functions significant enriched in AD samples. Only 2% of differentially expressed transcripts, mostly enriched in synaptic transmission functions, were shared between the vulnerable AD and PD regions, implying a complex and disease-specific microglial response.

Uriarte Huarte et al. (2021) recently examined microglial heterogeneity in the PD-susceptible SN region of non-diseased 6-month-old mice using a combination of in situ morphological analysis and single-cell transcriptomics [356]. Compared to microglia in the cortex and striatum, microglia in the midbrain region exhibited a distinct morphology with reduced ramification and branching, indicative of an activated state. A subset of microglia in the midbrain displayed a unique ‘immune-alerted’ transcriptional profile. The identified microglia cluster shares ~50% transcriptional similarity to reactive microglia under inflammatory conditions, including enrichment in processes associated with antigen presentation, cytokine signaling, and inflammatory responses, as well as inflammation-related biological pathways such as TLR, TNF, and NFκB pathways. In addition to immune-related profiles, this ‘immune-alerted’ microglia population also exhibited altered expression of genes essential for autophagy and ROS-related neurotoxicity. While this study did not examine microglia in a PD model, its findings highlighted the region-specific complexity of microglia, which may aid in elucidating potential mechanisms for the onset of PD.

More recently, Smajic et al. (2021) employed immunolabeling and single nuclei RNA-seq approaches to characterize the cell-specific response of cells in the midbrain of postmortem human PD tissue [357]. An increase in the number of microglia and a morphological change toward the amoeboid phenotype were evident in the SN of PD samples. Additionally, microglia in the PD brain exhibited a pro-inflammatory phenotype characterized by upregulation of Glycoprotein Nmb (GPNMB), Heat Shock Protein 90α (HSP90AA1) and IL-1β. Of the seven identified microglia groups, three main microglia subpopulations were highlighted, representing various microglial activation responses. P2RY12^high^ microglia cells were identified as resting subpopulations, while two activation branches were evident: one comprising GPNMB^high^ cells and another subpopulation containing cells with upregulated expression of HSP90AA1 or IL-1Β expression. Further characterization of the molecular phenotypes in PD revealed that the two activated subpopulations are enriched in cytokine signaling and unfolded protein response (UPR) pathways, and various PD risk factor genes linked to microglia activation profile are also present in these clusters, such as LRRK2 [357]. Upregulation of genes encoding heat shock proteins, which facilitate neuroinflammatory responses by acting as DAMPs, was also evident in the disease trajectory. Despite the limited knowledge of microglia phenotypes in PD, the highlighted transcriptome studies using scRNA-seq technology demonstrated that microglial responses in PD are heterogeneous and cannot be represented by the traditional M1/M2 paradigm. Additional studies of human microglia with a large sample size are needed to better characterize the temporal and regional responses of microglia at various stages of PD.

## 5. Modulators of Microglia Phenotypes in NDs

**Figure 3:**
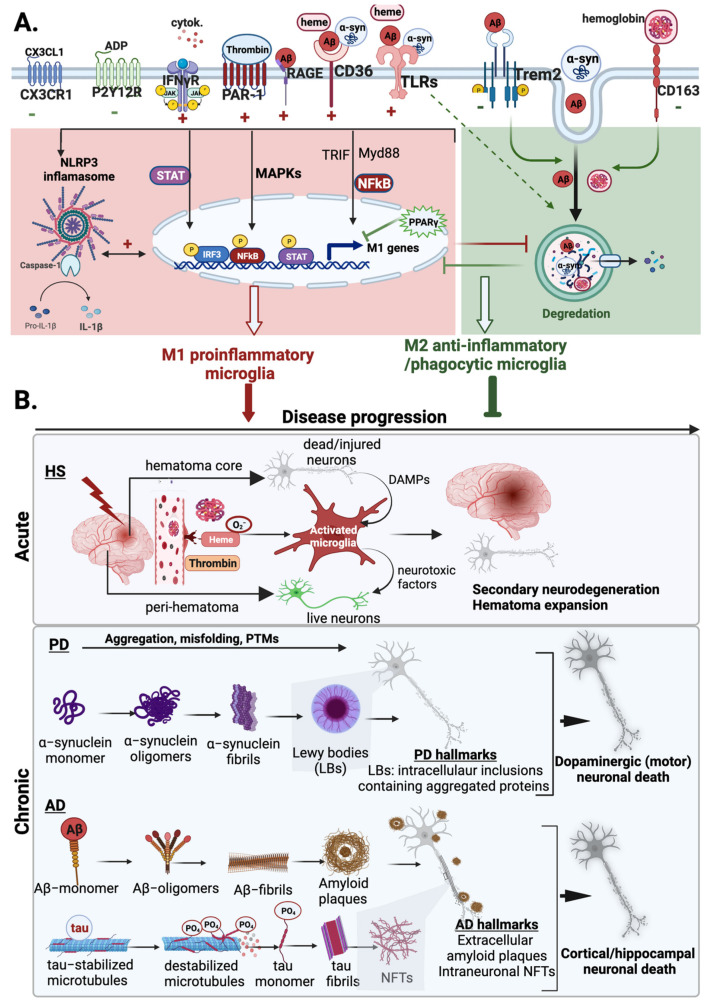
Microglial functions and diverse activation responses in neurodegenerative diseases. (**A**) Summary of the major activation mediators and receptors involved in microglial responses to Alzheimer’s disease (AD), Parkinson’s disease (PD), and hemorrhagic stroke (HS). Microglia can be activated directly by disease-specific triggers such as amyloid-β (Aβ) in AD, α-synuclein (α-syn) in PD, or hematoma products such as heme/hemoglobin and thrombin in HS. Various shared microglial receptors interact with these pathogenic molecules, and their overall response depends on the nature of the disease, the stage of the disease, and the specific molecular trigger. Microglial response is also modulated by other mediators such as cytokines present in the cerebrospinal fluid, damage-associated molecules (DAMPs), such as ADP, released from injured neurons, and neuron-derived endogenous signals such as CX3CL1. In general, microglial response can be pro-inflammatory/neurotoxic (+) or anti-inflammatory/neuroprotective (**−**). Activation of PAR-1, RAGE, CD36, and TLR receptors generally promotes pro-inflammatory responses by activating a range of pro-inflammatory pathways, such as JAK/STAT, MAPK, or NFκB, as well as the NLRP3 inflammasome. These diverse pro-inflammatory pathways can also interact synergistically, promoting chronic neuroinflammation. In contrast, activation of P2YR12 and CX3CR1 negatively regulates microglial activation and pro-inflammatory responses and generally mediates protective functions. Similarly, activation of TREM2 and other scavenger receptors induces microglial phagocytosis of Aβ and other pathogenic proteins, aiding in their clearance. These two generally distinct microglial responses also antagonize one another; degradation of abnormal proteins and pathogenic mediators helps limit chronic neuroinflammation, while pro-inflammatory responses can compromise the microglial phagocytosis response. (**B**) Summary of the role of microglia phenotypes in the progression of neurodegenerative diseases. In HS, microglia can be activated by thrombin or heme or indirectly by DAMPs from injured neurons, and a sustained microglial pro-inflammatory response contributes to secondary neurodegeneration in areas outside the initial injury zone. Pro-inflammatory microglial responses in PD and AD trigger post-translational modifications (PTMs) and misfolding and aggregation of α-syn and Aβ, that can contribute to the formation of amyloid plaques and Lewy bodies, thereby contributing to disease progression. Pro-inflammatory microglia also induce tau phosphorylation and subsequent aggregation, which promotes neurofibrillary tangle formation (NFT) and overall disease progression. Furthermore, pro-inflammatory microglia exhibit a dysregulated phagocytosis response, limiting the clearance of hematoma products and misfolded proteins that contribute to disease progression. On the contrary, anti-inflammatory/phagocytic microglia phenotypes mediate protective roles and limit disease progression by promoting tissue recovery. Figure created using BioRender.com (accessed on 14 April 2022).

As previously described, microglia in aging and NDs are highly plastic and can adopt a variety of activation phenotypes, often accompanied by an augmented inflammatory response and impaired homeostatic functions. There is considerable interest in identifying therapeutic targets that modulate microglia activity and promote the protective anti-inflammatory/phagocytic phenotype while suppressing pro-inflammatory and neurotoxic responses. This section will summarize a few therapeutically relevant receptors, signaling molecules, and pathways that have been implicated in modulating microglial phenotypes in a variety of pathological contexts (Figure 3). Apart from the targets discussed here, additional microglia modulators have been reviewed elsewhere [56,116], including many surface receptors (CD200R, CD36, prostaglandin E2 receptors) and key signaling molecules (PI3K/AKT, AMP-associated protein kinases (AMPKs), glycogen synthase kinase-3β (GSK3β), Rho-associated protein kinase (ROCK), and high-mobility group box-1 (HMGB1)).

### 5.1. TLR Signaling Pathway

Toll-like receptors (TLRs) are transmembrane pattern recognition receptors expressed on a wide variety of cells. TLRs are particularly abundant in microglia and their activity is dynamically regulated by different signals, including secreted endogenous DAMPs or exogenous PAMPs, such as bacterial endotoxin LPS [358,359]. Signaling mediated by these receptors, particularly TLR2 and TLR4, is one of the most extensively studied mechanisms for inducing classical microglia activation and neuroinflammation [167]. Both TLR2 and TLR4 signal via receptor dimerization and subsequent recruitment of the adapter protein Myd88 to activate the pro-inflammatory NFκB pathway; TLR4 can alternatively signal via a Myd88-independent mechanism involving the TRIF adaptor [358,359].

Activation of the TLR/Myd88/NFκB pathway results in the production of pro-inflammatory cytokines, chemokines, and ROS/RNS species, thereby enhancing neuroinflammation and neurotoxicity [167]. TLR4 activation also triggers other pro-inflammatory cascades such as mitogen-associated protein kinases (MAPKs) pathways [360]. TLR4 activation is observed in many NDs, including AD, PD, and both hemorrhagic stroke subtypes (ICH and SAH), and its upregulation is associated with a pro-inflammatory response and adverse outcomes [116,167,181,270]. High TLR4 expression is correlated with cerebral vasospasm, ischemia, and neuronal injury in patients with SAH [168], and its expression in ICH alters the clearance of hematoma and aggravates neurological deficit [170,181]. TLRs serve as receptors for Aβ and α-syn in AD and PD, respectively, and their activation can promote pro-inflammatory and neurotoxic responses [270].

Significant evidence in experimental disease models suggests that TLR4 or Myd88 deficiency provides neuroprotective effects by promoting the switch of microglia from the M1-like to M2-like phenotype and limiting microgliosis [361,362]. Similarly, NFκB activation plays a role in a variety of NDs, and inhibition of this pathway limits pro-inflammatory responses and promotes a protective M2 phenotype [116,363]. Therefore, therapeutic strategies that block the TLR4 pathway, such as astaxanthin and peroxiredoxin 2, have been explored in various experimental models. In an ICH model, TLR4 antagonism or genetic deletion showed neuroprotection by limiting microglial activation in perihematomal tissue [364,365]. Similarly, inhibition of TLR4 induces M2 polarization and provides neuroprotection in AD model [366].

Conflicting evidence suggests that TLR4 plays a protective role in various NDs models, due to its role in enhancing microglial phagocytosis of pathogenic proteins [367]. TLR4-mediated activation of microglia in the early stage of AD protects neurons by limiting Aβ deposition [368]; however, sustained Aβ exposure and TLR4 activation during disease progression alters microglial response by promoting a switch from the phagocytic phenotype to a neurotoxic phenotype [367,369]. In addition to TLR4, TLR2 is also implicated in NDs, and activation of this receptor has been shown to induce microglial response and exacerbate neuronal injury in models of cerebral stroke, AD, and PD [359]. However, its activation in a spinal injury model was shown to be protective by promoting a switch of microglia phenotype from M1-like to an alternative M1:M2 phenotype [370]. Therefore, the precise role of TLRs in modulating microglial polarization or injury outcome is unclear and varies according to the specific pathological condition, activating signals, and the disease stage.

### 5.2. JAK/STAT Pathway

Another critical pathway involved in pathogenic inflammatory responses is the Janus kinase (JAK)-STAT signaling cascade. JAK-STAT signaling is important in modulating innate and adaptive immune responses, and abnormal activation of this pathway is observed in multiple NDs [371]. JAK/STAT signaling is activated by binding of cytokines and interferons to their cognate receptors. Receptor-associated activation of JAKs induces the phosphorylation and subsequent nuclear translocation of STAT transcription factors, which promote the expression of inflammatory genes [371]. JAK/STAT signaling has been shown to modulate microglia phenotypes in vitro and in vivo. STAT1 promotes M1 polarization in response to hypoxic stimuli by upregulating CD86, COX-2, and iNOS [372]. In vivo, inhibition of JAK/STAT reduces M1 polarization while increasing M2 polarization [373,374,375]. JAK/STAT signaling is also involved in the regulation of microglial responses in pathological conditions. For example, SAH can trigger immediate phosphorylation and nuclear translocation of STAT3 and JAK/STAT3 inhibitors, such as erythropoietin and AG490, limiting M1 pro-inflammatory responses and improving brain recovery [376,377]. Similarly, inhibition of the JAK/STAT pathway in PD prevented neuroinflammation and neurotoxicity by limiting a-syn-induced microglial pro-inflammatory mediators [378]. These findings suggest that inhibition of microglial JAK/STAT signaling may have therapeutic potential for NDs.

### 5.3. CX3CR1-CX3CL1 Signaling

The CX3C chemokine receptor 1 (CX3CR1) and the fractalkine ligand (CX3CL1) form another important signaling axis that regulates microglial activation and function. CX3CR1 is expressed primarily by microglia and interacts with its neuron-secreted CX3CL1 ligand to mediate reciprocal microglia-neuron crosstalk, which serves as an important immune checkpoint [18]. For example, the interaction of CX3CR1 and CX3CL1 suppresses microglial M1 pro-inflammatory responses such as the expression of IL-1β, IL-6, TNFα, and iNOS [379,380,381], and downregulation of either CX3CR1 or CX3CL1 is associated with enhanced microglial activation and neuroinflammation [382]. CX3CR1-CX3CL1 signaling has been shown to be neuroprotective in a variety of neurodegenerative models [18]. For example, dysregulated CX3CR1-CX3CL1 signaling enhances microglial activation and promotes DA neuronal degeneration in PD [380,383,384]. In both neurotoxic and α-syn PD models, overexpression of CX3CR1 or exogenous administration of CX3CL1 protected against microglia-mediated neurotoxicity [385,386]. A recent study showed that CX3CR1 knockout young microglia exhibit a premature aging transcriptome, accompanied by an age-associated alteration in microglial morphology and upregulation of inflammatory of inflammatory pathways [387]. These findings implicate a protective role for CX3CR1-CX3CL1 in suppressing neurotoxic microglial activation during aging. Interestingly, CX3CR1 has been associated with neurotoxic and neuroprotective functions in the AD model, and its diverse role in AD has been attributed in part to the competitive interaction of CX3CR1 with its endogenous ligand CX3CL1 or its pathological ligand tau, which can differentially affect microglial phagocytosis or activation response [18,245]. Overall, the CX3CR1-CXCL1 signaling modulates microglial inflammatory responses, and therapeutic modulation of these molecules should take into account the specific pathological insult and the disease stage.

### 5.4. Triggering Receptors Expressed on Myeloid Cells 2 (TREM2)

Triggering receptors expressed on myeloid cells 2 (TREM2) is a cell surface receptor that is expressed in myeloid cells and participates in various innate immune responses [197]. In the CNS, TREM2 and its adaptor protein DNAX-activation protein 12 (DAP12) are enriched in microglia, and activation of this complex regulates pro-inflammatory and phagocytosis responses [197,388]. TREM2 also mediates various protective functions, including sensing, housekeeping, and host defense responses [270]. TREM2 recognizes damage-associated lipid substrates such as phosphatidylserine expressed on the surface of apoptotic neurons, and it also binds ligands such as apoprotein E (apoE) and Aβ, subsequently eliciting chemotactic and phagocytic responses that ultimately mediate the clearance of apoptotic neurons and pathogenic proteins [197,389,390].

TREM2 was recently identified as an important receptor that regulates the microglial phenotypic switch from inflammatory M1 microglia to the immunosuppressive M2 phenotype [391]. TREM2 knockdown in BV2 cells upregulates M1 microglial inflammatory responses and inhibits M2 polarization, while overexpression reverses this phenotype. In the absence of TREM2, the M2 polarizing cytokines, IL-4 and IL-13, cannot induce the expression of the M2 marker, indicating that TREM2 is required for adequate M2 polarization [391]. The expression of TREM2 is also downregulated by pro-inflammatory signals [392], which can further promote chronic neuroinflammation.

TREM2 also modulates microglial function in disease states, and several studies demonstrated that TREM2 deficiency impairs the clearance of apoptotic neurons and pathological substrates, thus attenuating disease progression [197,327,390]. TREM2 inhibition promotes pro-inflammatory microglial response and neurological deficit in a HS model [393]. Microglial TREM2 is also strongly implicated in AD pathology, and loss of its expression alters microglial phagocytosis responses, which contribute to the formation of dispersed Aβ plaques associated with increased neurotic damage [201]. However, contradictory findings indicate that TREM2 may also play a detrimental role in AD pathology, suggesting that TREM2 functions differently depending on the stage of disease progression. For example, Sheng et al. (2019) demonstrated a dual role for TREM2 in different stages of APP/PS1 mice [394]. The phagocytic function of microglial TREM2 in the early middle pathological stage (2–6 months old mice) exacerbated AD pathology by inducing synaptic loss, while its expression in advanced stages (6–10 months) prevented amyloidosis by limiting amyloid deposition. Therefore, the multifaceted function of TREM2 and its contradictory role in disease models warrant additional longitudinal research to determine its therapeutic potential.

### 5.5. P2Y Receptors

Purinergic P2Y receptors are G-protein-coupled receptors that are activated by endogenous DAMPs such as nucleotides and play an essential role in modulating microglial response to stress and injury [395]. Recent research in animal models and human tissues has implicated the P2Y6 receptor (P2Y6R) and the P2Y12 receptor (P2Y12R) in the pathogenesis of multiple NDs. P2Y6R is essential for microglial phagocytosis of neurons and is primarily activated by UDP released by stressed/injured neurons [109]. P2Y6R also facilitates microglial clearance of amyloid plaques and improves cognitive defects in AD [396]. Additionally, P2Y6R-mediated phagocytosis mediates the removal of neuronal debris in a cerebral stroke model [397]. Although these findings demonstrate the protective role of P2Y6R, microglial phagocytosis response induced by this receptor has been shown to exacerbate AD pathology by promoting microglial phagocytosis of stressed but viable neurons, which are capable of releasing UDP [398]. In mixed neuron/glia culture, the use of the P2Y6R antagonist MRS2578 delayed the neuronal loss induced by Aβ, and this effect was mediated by inhibiting microglial phagocytosis response. Similarly, mice lacking P2Y6R were protected from neuronal loss and cognitive deficit caused by aging and Aβ/tau pathology [398,399]. P2Y6R is also implicated in PD pathology and has been considered a potential PD biomarker due to its high expression in patients [400]. The role of P2Y6R in PD is unclear, but has been implicated in enhancing neuroinflammatory responses [400]. These studies highlighted the multifaceted role of UDP/P2Y6R signaling in mediating microglia-induced neurodegeneration.

The P2Y12 receptor is expressed exclusively in microglia in the CNS and is activated primarily by ADP, which is derived from the breakdown of ATP released from neurons and glia cells [395]. P2Y12R regulates microglial activation and migration and is strongly involved in the early stage response of microglia in NDs [278,401,402,403]. Recent scRNA-seq studies in various ND models identified P2Y12R-expressing microglia as a resting microglia subpopulation with crucial housekeeping and defense functions [278]. Downregulation of P2Y12R was shown to be one of the key alterations in signature transcripts as microglia transition from a homeostatic state to a neurotoxic phenotype [278,403]. Diminished expression of P2Y12R has been evident in models of AD and other tauopathies, and P2Y12R-deficient microglia are found closely in regions of the brain with dense tau aggregates and significant neurodegeneration [402]. The expression level of P2Y12 also declines with age, which may contribute to age-associated changes in the microglial activation response [95]. Overall, these findings suggest that rescuing the loss of P2Y12R during aging may have a potential therapeutic effect for NDs. However, additional research is necessary to investigate the mechanism and functions of P2Y6R and P2Y12R in different pathological conditions and disease stages.

### 5.6. Peroxisome Proliferator-Activated Receptor γ (PPARγ)

The peroxisome proliferator-activated receptor γ (PPARγ), a highly expressed nuclear receptor in microglia, is a ligand-activated transcription factor that plays an important role in immune responses [404]. Preclinical evidence suggests that PPARγ activation exerts neuroprotective effects in response to harmful stimuli and induces a phenotypic switch from M1 to M2 microglia [405,406,407,408]. Given the anti-inflammatory and neuroprotective functions of PPARγ, the FDA-approved antidiabetic drugs rosiglitazone and pioglitazone, which function as agonists for PPARγ, have been considered for the treatment of NDs [409]. Experimental studies in stroke models demonstrated that agonist-induced PPARγ activation promotes the expression of M2 markers (CD206, YM-1, IL-10, and TGF-β) while inhibiting classic M1 markers (iNOS, CD16, CD32, CD86, MMP-9) [406,407,408].

PPARγ also plays a protective role in AD and PD. In a PD model, administration of PPARγ agonists reduced MPTP-induced pro-inflammatory cytokine production and prevents DA neuronal loss in the SN [410,411]. Similarly, PPARγ agonists promote a phenotypic switch from the M1 to M2 phenotype in AD mice and enhance microglial amyloid clearance by upregulating microglial scavenger receptors such as CD36 [57,405,412]. PPARγ agonist improves the uptake of misfolded protein deposits in AD mice, thereby suppressing Aβ levels and rescuing cognitive function [412]. In a preclinical ICH model, PPARγ agonist improved hematoma clearance, neuronal injury, and functional recovery [413], and in vitro, PPARγ activation induces upregulation of CD36 and facilitates microglial phagocytosis of red blood cells [414]. Due to the beneficial role of PPARγ in modulating microglial function, several clinical trials have been initiated to evaluate the therapeutic potential of pioglitazone in NDs.

## 6. Challenges in Microglia Research

### 6.1. Specific Microglia Markers

Neuroscience research on microglia commonly employs a variety of techniques, including immunohistochemistry, microglia depletion method, in vivo imaging, and cre-lox systems [415]. These technologies rely on markers to detect and/or target microglia, such as CX3CR1, CSF1R, Cd11b, and Iba1. However, commonly used microglia markers, particularly those that identify an activated state, do not differentiate resident microglia from infiltrating monocytes that enter the brain under pathological conditions characterized by a compromised blood–brain barrier [416]. This lack of specificity is a significant impediment to microglia research, as most studies are limited to examining a mixed microglia/macrophage population. Flow cytometry enables a more selective separation of microglia cells that exhibit CD11b expression and low-to-intermediate levels of CD45 (CD11b^+^, CD45^low-to-intermediate)^, from a macrophage population with high expression of CD45 (CD11b^+^, CD45^high^) [417]. However, this approach has been ineffective in pathological models, since microglial CD45 expression is upregulated in response to inflammation [418]. Recently, unique microglial markers such as P2Y12R and Tmem119 have been identified [13,14]. However, the utility of these putative markers in disease models has not been thoroughly investigated, and there are reports of decreased expression of these receptors under pathological conditions [419], which may present a hurdle to targeting microglia in disease models.

### 6.2. Sex-Specific Heterogeneity of Microglia

Another major hurdle in microglia research is the sex-associated differences in microglial responses. Microglia from male and female brains exhibit different characteristics, including differences in cell morphology, density, transcriptional profile, and cellular functions [420]. Male microglia are characterized by a larger soma size, a higher migration capacity, increased pro-inflammatory gene expressions, and a shorter lifespan, while female microglia have enhanced phagocytic capacity accompanied by increased expression of scavenger receptors, and have higher expression of protective genes involved in immune regulation and cellular repair [421,422,423]. Given the central role of microglial phagocytosis and inflammation in NDs, gender-specific differences in microglial response should be carefully examined to elucidate disease mechanisms that contribute to sex bias in susceptibility to aging and NDs (refer to Yanguas-Casas, 2020 for a detailed review [420]). 

### 6.3. Limitation and Heterogeneity of Human Microglia

Another complication in aging studies arises from species-specific heterogeneity of microglia. Despite the significant utility of animal models, there are inherent differences between human and rodent microglia, most notably in the aged brain, that can influence how microglia respond in NDs. Recently, a major distinction between human and mouse microglia was reported, demonstrating that human microglia have a unique transcriptional signature, including exclusive expression of cell cycle and proliferation genes, as well as signature genes involved in the complement system and neurodegeneration [424]. Human microglia exhibited age-dependent upregulation of genes associated with immune functions and pro-inflammatory responses, suggesting a “preactivated” state. In the presence of a disease-specific environment, already primed human microglia can foster a sustained inflammatory response. Furthermore, human microglia have been shown to exhibit greater heterogeneity in the transcriptome than microglia from animal models [424].

Since microglial activation and function are influenced by the natural aging process, commonly used preclinical models, such as transgenic animals that harbor risk factor alleles or genetic mutations, may not accurately mimic the response of microglia to human NDs. These factors, in addition to age-associated interspecies microglial differences, strongly support the need for human microglia research, which unfortunately has its own limitations. Obtaining primary human microglia for in vitro research is highly invasive, and the ‘preactivated’ nature of human microglia and its dynamic plasticity may preclude their use in vitro cultures, as changes in the microenvironment can significantly alter microglial transcriptome signatures and function [425]. Recent research has shown that when microglia obtained from postmortem tissue are cultured in vitro, their gene expression profile significantly changes, including loss of microglia-specific markers, indicating the need for rapid microglia phenotyping [426]. In addition to the influence of postmortem delay, human postmortem findings have limitations in that extraneous agonal factors, such as the manner and terminal state before death, have been found to alter postmortem brain gene expression [427]. Therefore, when interpreting gene expression data from postmortem brains, confounding variables that affect microglia profiling must be carefully adjusted during data analysis.

**Figure 4:**
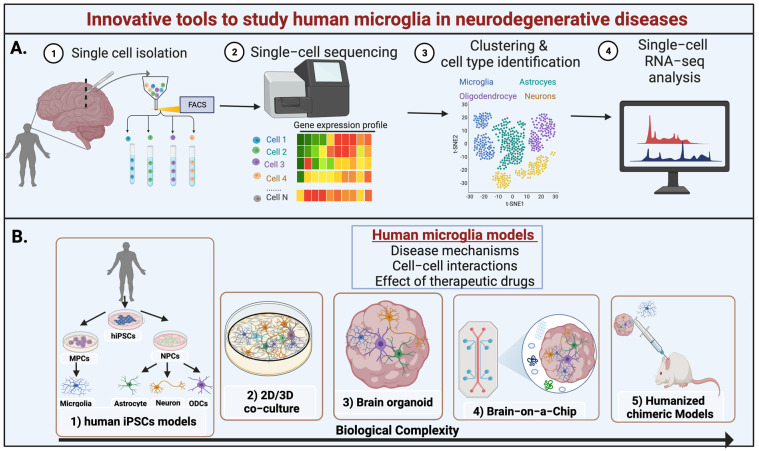
Innovative tools to study human microglia in neurodegenerative diseases. (**A**) Single-cell sequencing can be performed by isolating single cells from live or postmortem brains. This tool assists in characterizing the gene expression profiles of various cell types, which helps to understand the heterogeneous phenotypes of microglia in human diseases. (**B**) The generation of microglia-like cells from human-induced pluripotent stem cells (hiPSCs) has advanced mechanistic studies. iPSC can be derived from patients with neurodegenerative diseases or can be genetically modified to study human microglia responses in a specific disease environment. iPSC-derived microglia can also be co-cultured in two/three-dimensional systems or incorporated into brain organoids to study complex interactions of microglia with neurons and other cells. Brain-on-a-Chip technology employs a continuous flow of microscopic fluid in organoid systems, which enables the addition of extracellular matrix components to mimic the complex properties of the brain or incorporates pathogenic stimuli to emulate disease-specific brain microenvironment. Humanized microglia models, created through xenotransplantation of human iPSC-derived microglia models, help to study human microglia in intact models, and this technology is beneficial for translational research, such as therapeutic drug testing. Figure created using BioRender.com (accessed on 14 April 2022).

Given these limitations, recent tools have been explored for large-scale studies of human microglia (Figure 4). Single-cell sequencing has become a powerful method to effectively capture the cellular heterogeneity of the brain and has helped characterize heterogeneous microglia subpopulations in the human brain using limited starting material. Additionally, the generation of microglia-like cells from human-induced pluripotent stem cells (hiPSCs) enables researchers to conduct mechanistic studies that require a large supply of human cells [428]. hiPSC-derived microglia also have the advantage in that they can be derived from neurodegenerative patients or genetically modified to mimic specific pathological conditions, allowing researchers to study heterogeneous microglial responses in the context of human disease.

Incorporation of iPSC-derived microglia into a 3D cerebral organoid model also provides a great opportunity to study the complex interaction of microglia with neurons and other glial cells [429]. One major technical challenge in using organoid models is the fact that cell culture conditions may not permit simultaneous growth of different cell types, making it difficult to maintain the physiological ratio and distribution of cells in the brain. Recently, a cell-based microfluidics system was engineered, such as Brain-on-a-Chip technology, allowing researchers to incorporate organoid models in a continuous flow of microscopic fluid [430]. This approach can help mimic the complex structural and functional properties of brain tissue by integrating components of the extracellular matrix. In addition, pathological stimuli, such as amyloid-β or α-synuclein, can be added to the microfluidic system to emulate disease-specific brain microenvironments. Finally, humanized microglia models have been developed by xenotransplanting human iPSC-derived microglia into immunodeficient mice expressing human microglia growth factors such as human CSF1 to maintain survival [284,431,432]. Interestingly, compared to human iPSC-derived microglia, the transcriptional profile of microglia from a humanized model resembled human microglia more closely [431,432]. Overall, the use of multiomics technologies and innovative experimental models, such as organoid cultures and humanized chimeric mice, can provide powerful systems for studying the complex response of human microglia during aging and in NDs (Figure 4).

## 7. Conclusions and Outlook

This paper reviewed existing knowledge on the diverse functions and phenotypes of microglia in health, aging, and neurodegenerative diseases (NDs), highlighting the crucial role of microglia in the onset and progression of hemorrhagic stroke (HS), Alzheimer’s disease (AD), and Parkinson’s disease (PD). HS is an acute vascular disorder that causes neurodegeneration, while AD and PD are progressive chronic NDs with complex etiologies. PD selectively affects midbrain dopaminergic motor neurons in the substantia nigra and is commonly associated with motor deficit, while AD has a more widespread effect on hippocampal and cortical neurons and primarily affects cognitive functions. These three neurodegenerative conditions share pathogenic mechanisms associated with age, such as inflammation and oxidative stress, in which activated microglia play an important role.

Microglia can be activated by blood-derived components such as heme/thrombin in the case of HS, by misfolded α-synuclein aggregates in PD, and by extracellular amyloid-β and/or intraneuronal phosphorylated tau in AD. Microglial phagocytosis and activation in response to these mediators are facilitated through the activation of shared receptors, such as TLRs and scavenger receptors, which can lead to activation of various inflammatory pathways, including the NF_k_B, JAK-STAT, and NLRP3 inflammasome (Figure 3). Microglia-mediated uptake of heme, α-syn, and Aβ/tau can also have protective effects by removing these pathogenic mediators from the extracellular space. However, as disease progresses, a chronic inflammatory and oxidative environment can promote sustained microglial activation that can cause neuroinflammation, oxidative stress, iron overload, and neurotoxicity. Microglia can also be activated by secondary mediators such as nucleotides, complement proteins, cytokines, and matrix metalloproteinases.

Microglial response is complex and is shaped by the underlying pathology, which depends on the nature of the disease and the specific activation trigger. For example, microglia can respond differently based on the chemical and structural properties of pathogenic proteins. Post-translationally modified or aggregated forms of Aβ and α-syn tend to provoke a more neurotoxic microglial response compared to unmodified or monomeric forms. Furthermore, regional cues differentially influence the activation state and functional response of microglia. For example, unlike intraneuronal tau molecules, extracellularly deposited Aβ has a greater propensity to activate and recruit peripheral cells, and these cells can subsequently modulate microglial response, contributing to the existence of diverse microglial phenotypes.

The general response of microglia has traditionally been simplified into two phenotypic states: an M1-like pro-inflammatory phenotype associated with neurotoxicity, and an M2-like anti-inflammatory and/or phagocytic phenotype involved in the clearance of hematoma/misfolded proteins, the resolution of inflammation, and tissue recovery. However, microglial response in NDs is highly dynamic and has diverse activation profiles. As disease progresses, the balance between protective and neurotoxic responses changes, and a dominant microglia phenotype can exist at a specific disease stage. Thus, targeting microglial M1/M2 balance and promoting a phenotypic switch from a pro-inflammatory phenotype to a protective phenotype have great therapeutic potential, and this can be achieved using immunomodulators that activate TREM2, PPARγ, and CX3CR1-CX3CL1 signaling, or by inhibiting TLR/NF_k_B and JAK/STAT pathways (Figure 3).

The specific type of disease and the stages of clinical progression can also play a role in modulating microglia phenotypes. For example, in HS, rapid hematoma formation induces acute M1-like polarization, and the M1/M2 balance decreases over time, with the M2-like phenotype serving a protective role in the recovery phase. This polarization trajectory appears to be reversed in AD, with a protective M2-like phenotype predominating in the early stages of AD and a pro-inflammatory phenotype emerging as the disease progresses and Aβ clearance fails. Therefore, therapeutic interventions should consider the stage-specific microglial response. In HS, inhibiting the M1-like microglia response in the acute stage and promoting an early transition to the protective M2 phenotype may be beneficial, while in AD, immunomodulators that stabilize the protective microglia phenotype in the early/presymptomatic phase, before their transition to a neurotoxic phenotype, may be beneficial.

Since age is a shared risk factor for all three diseases, it may be beneficial to define and target preventive mechanisms that predispose people to NDs, such as age-dependent microglial alterations (Figure 2). Microglial priming and/or dysfunction are triggered by aging and are characterized by increased M1/M2 balance, impaired calcium homeostasis, and loss of sensing and phagocytosis responses. Defect in microglial phagocytosis limits the clearance of cellular debris, misfolded proteins (AD and PD), and hematoma elements (HS), which can trigger the onset and progression of NDs. Therefore, immunomodulators capable of restoring age-related loss of immunoregulatory proteins, such as TGF-β and CX3CL1, or those that block upregulated pro-inflammatory receptors, such as TLR4 and MHCII, have potential preventive roles. Furthermore, complement-mediated clearance mechanisms that are reactivated in the aged brain could be targeted to prevent age-related synaptic loss. Therefore, rescuing age-dependent microglia alterations may be beneficial to prevent the onset of NDs.

In conclusion, a detailed and longitudinal characterization of microglial phenotypes during aging, in presymptomatic and symptomatic individuals, is necessary to identify an optimal disease-specific therapeutic window. Tremendous developments such as single-cell transcriptomics, innovative in vivo imaging technologies, humanized microglia models, and 3D organoid and Brain-on-a-Chip models are advancing mechanistic and translational microglia research, which will help elucidate the dynamic activation and functional changes of microglia in NDs.
